# “The NET effect”: Neutrophil extracellular traps—a potential key component of the dysregulated host immune response in sepsis

**DOI:** 10.1186/s13054-025-05283-0

**Published:** 2025-02-04

**Authors:** Andrew Retter, Mervyn Singer, Djillali Annane

**Affiliations:** 1 Critical Care, Guy’s and St Thomas’ NHS Foundation Trust, London, UK; 2 School of Immunology and Microbial Sciences, King’s College, London, UK; 3 Volition, London, UK; 4 Bloomsbury Institute of Intensive Care Medicine, Division of Medicine, University College London, London, UK; 5 Department of Intensive Care, Raymond Poincaré Hospital, APHP University Versailles Saint Quentin–University Paris Saclay, INSERM, Garches, France; 6 IHU PROMETHEUS, Comprehensive Sepsis Center, Garches, France; 7 University Versailles Saint Quentin–University Paris Saclay, INSERM, Garches, France; 8 FHU SEPSIS (Saclay and Paris Seine Nord Endeavour to PerSonalize Interventions for Sepsis), Garches, France

**Keywords:** Neutrophil extracellular traps (NETs), Sepsis, Immunothrombosis, Thromboinflammation, Histones, Complement, DAMPs (damage-associated molecular patterns), Coagulation, NETosis, Inflammation, Innate immune response, Host immune response, Thrombosis

## Abstract

Neutrophils release neutrophil extracellular traps (NETs) as part of a healthy host immune response. NETs physically trap and kill pathogens as well as activating and facilitating crosstalk between immune cells and complement. Excessive or inadequately resolved NETs are implicated in the underlying pathophysiology of sepsis and other inflammatory diseases, including amplification of the inflammatory response and inducing thrombotic complications. Here, we review the growing evidence implicating neutrophils and NETs as central players in the dysregulated host immune response. We discuss potential strategies for modifying NETs to improve patient outcomes and the need for careful patient selection.

## Background

Sepsis is characterised by a dysregulated host response to infection, leading to life-threatening organ dysfunction [1, 2]. With an estimated global incidence of 49 million cases and 11 million deaths annually, sepsis represents a significant public health challenge [2, 3]. Recent advancements in our understanding of sepsis immunobiology have led to a more nuanced conceptualisation, incorporating immune-driven resistance, tolerance, resilience, resolution and repair [4].

Central to this evolving paradigm is the concept of immunothrombosis, a host defence mechanism that integrates the immune and coagulation systems to contain and eliminate pathogens in the bloodstream [5, 6]. This process involves the coordinated activation of multiple cellular and molecular components, including neutrophils, platelets, monocytes, the complement system, damage-associated molecular patterns (DAMPs), and coagulation factors [7, 8]. While immunothrombosis serves a protective role under normal circumstances, excessive or uncontrolled activation can lead to widespread thromboinflammation [8–11]. In addition, the role of DAMPs, host cellular constituents released from damaged or stressed cells, act as danger signals that trigger inflammatory responses through pattern recognition receptors (PRRs), such as Toll-like receptors (TLR) and NOD-like receptors [12, 13]. However, excess DAMPs can also play an important role in amplifying and perpetuating the inflammatory response in sepsis and directly contributing to organ dysfunction [12, 14].

Neutrophil extracellular traps (NETs) composed of extruded nuclear chromatin decorated with histones and granular proteins, serve to trap and neutralise pathogens [15]. However, excessive NET formation and/or dysregulated clearance have been implicated in exacerbating sepsis pathophysiology by promoting inflammation, tissue damage and thrombosis [16, 17]. The intricate balance between protective and detrimental properties underscores the complexity of the host response in sepsis. Understanding these mechanisms is crucial for developing targeted immunomodulatory therapies to improve patient outcomes [18]. This narrative review aims to:

1. Describe the growing evidence implicating neutrophils and NETs as central players in both host defence and the dysregulated immune response in sepsis
2. Explore the role of NETs in immunothrombosis and thromboinflammation
3. Discuss potential strategies for modifying NETs to improve patient outcomes.

By elucidating these complex interactions, we aim to provide insights into novel therapeutic approaches that can modulate the immune response and potentially mitigate tissue damage in sepsis and other systemic inflammatory conditions.

## The history and discovery of NETs

Neutrophils play a critical role in the innate immune response to any inflammatory insult. However, they present unique challenges to study due to their intrinsic characteristics and to technical issues related to their investigation [19]. Our understanding of neutrophil pathobiology consequently lags well behind that of other immune cells. Neutrophils have a short lifespan, typically surviving 5–7 days in circulation, that is reduced once activated [20]. This short lifespan is beneficial for supporting a rapid response to infection but presents a significant obstacle for researchers attempting to isolate cells and expand them in vitro. Once isolated from blood, neutrophils remain viable for only a few hours, limiting the window for experimental manipulation and analysis. The process of isolating neutrophils also inadvertently activates them, altering their physiology and potentially confounding results [21]. Neutrophil activation triggers a variety of processes including degranulation and production of reactive oxygen species (ROS) that can affect their function and interactions with other cell types [22, 23]. This sensitivity means that even minor changes in isolation and handling techniques can lead to significant variability in experimental outcomes. Neutrophils also require the presence of other cells, in particular monocytes and platelets, to facilitate their activation. This makes in vitro study more complicated and harder to achieve consistency in experiments [21, 24]. Neutrophils also exhibit a surprising degree of functional heterogeneity, with subsets displaying distinct phenotypic and functional profiles [25], the full extent of which is still being explored [23]. Such complexity adds another layer of investigative difficulty in view of the potential influence of these different subpopulations on experimental outcomes. Neutrophils perform many of their key functions within tissues and not just in the bloodstream. Studying these cells in their native context requires sophisticated techniques such as intravital microscopy that are not universally available [26]. Ethical and technical considerations also limit the extent to which invasive studies can be applied to humans to observe neutrophil behaviour under physiological and pathological conditions. Neutrophils only constitute 10–25% of circulating white cells in rodents [27], so direct transferability of findings is uncertain.

**Fig. 1:**
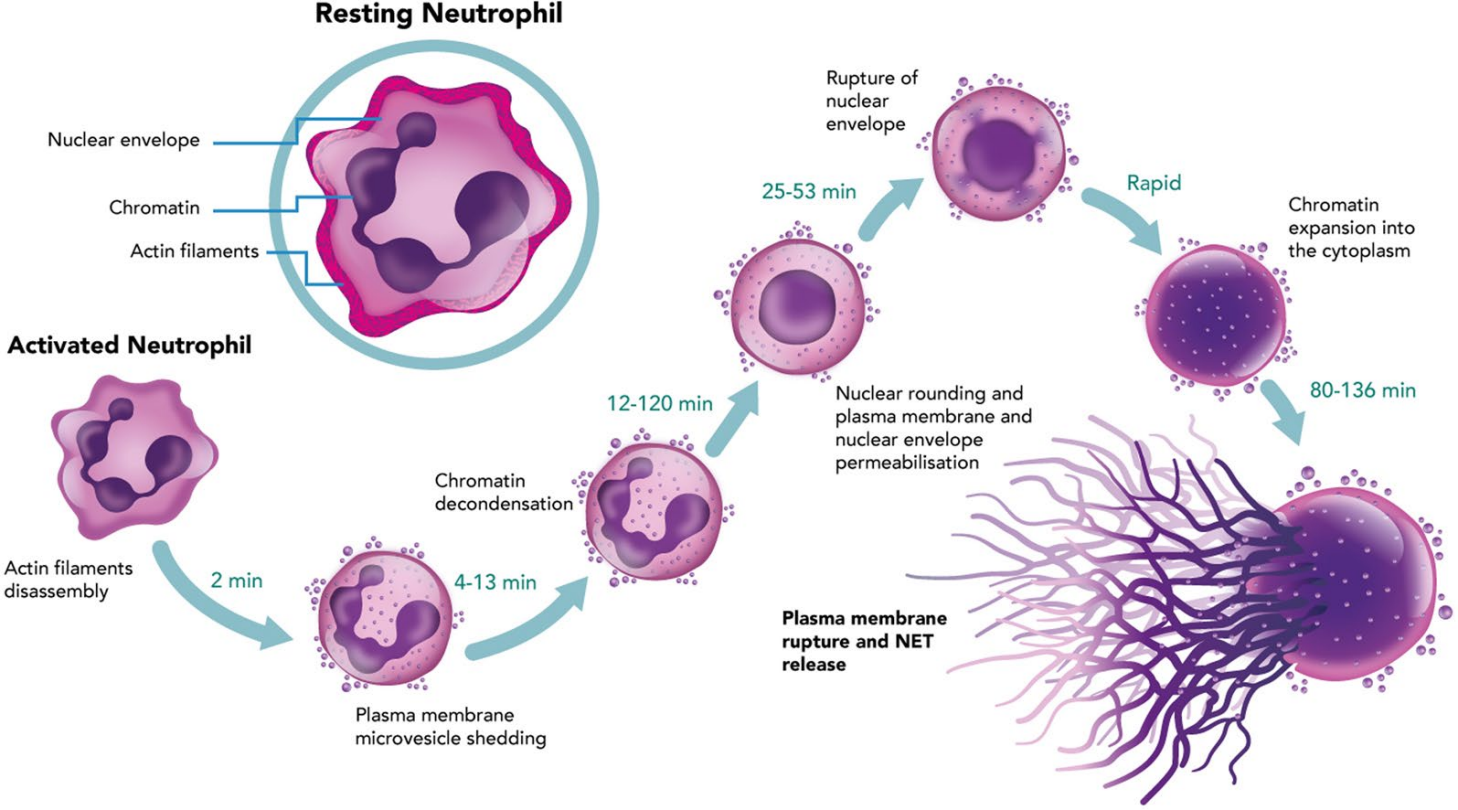
Timeline showing the formation and release of NETs from an activated neutrophil. *Min* minute, *NET* neutrophil extracellular trap

In 2004, Brinkmann et al. observed that activated neutrophils released their nuclear contents forming extracellular fibres that could trap and kill bacteria (Fig. 1) [15]. This observation was initially met with scepticism as it challenged the conventional view of neutrophils as short-lived, primarily killing pathogens through phagocytosis and degranulation [28]. In 2007, Fuchs et al. demonstrated that NET formation was triggered by a novel form of active cell death, NETosis, which required generation of ROS by nicotinamide adenine dinucleotide phosphate oxidase (NADPH oxidase, NOX), a key enzyme in the neutrophil respiratory burst [29]. NET release is induced by a wide range of stimuli, for instance, bacteria, viruses, fungi and parasites; pro-inflammatory mediators such as interleukin (IL)−8, lymphotoxin-alpha and tumour necrosis factor alpha (TNFα); platelets, activated endothelial cells, and components of the complement system [24, 30, 31].

NETs are composed of a mix of chromatin, histones, nucleosomes and granular-derived components such as neutrophil elastase (NE), myeloperoxidase (MPO) and cathepsin G [32, 33]. Both NE and cathepsin G are multifunctional neutrophil serine proteases with important roles in the inflammatory-immune response [33]. Their shared functions include: (i) antimicrobial properties that enable them to directly kill or inactivate pathogens [33, 34]; (ii) extracellular matrix degradation to facilitate cell migration and tissue remodelling during inflammation [33, 35]; (iii) neutrophil recruitment to sites of inflammation or infection by inducing neutrophil activation and chemotaxis; and (iv) proteolytical regulation of cytokines, chemokines and other inflammatory mediators [33, 36]. Cathepsin G also activates platelets, contributing to thrombosis [33, 36]. The heme-containing enzyme MPO is expressed by neutrophils and plays a crucial role in the innate immune response. It catalyses production of chlorinating oxidants, such as hypochlorous acid, facilitating oxidative killing of pathogens during phagocytosis [37, 38]. MPO can also modulate inflammation independent of its enzymatic properties by regulating neutrophil function and NET formation [37]. Extracellular histones can directly activate platelets, promoting their pro-inflammatory and pro-thrombotic functions [39]. In turn, activated platelets can trigger neutrophils to undergo NETosis [30], creating a positive feedback loop of inflammation and tissue damage.

The process of NETosis is pivotal for trapping and neutralising pathogens, preventing their spread, and facilitating their clearance [30]. However, dysregulated NET formation and clearance have been implicated in a broad spectrum of diseases, highlighting a paradoxical role in both defending against infection yet exacerbating disease pathology [40, 41]. Excessive or inadequately resolved NETs contribute to the pathology of chronic inflammatory and autoimmune diseases by promoting inflammation, tissue damage and thrombosis [42]. For example, high levels and increased activity of cathepsin G have been linked to the pathogenesis of rheumatoid arthritis and systemic lupus erythematosus (SLE) [36]; uncontrolled NET activity has been implicated in acute respiratory distress syndrome (ARDS) [43]; while elevated plasma levels of MPO are frequently detected in patients with sepsis [37]. In the context of sepsis, uncontrolled NET formation exacerbates endothelial damage and can promote microvascular thrombosis [5, 8]. The persistence of NET components can act as autoantigens, triggering autoimmune responses in susceptible individuals [44]. Targeting NETs and their regulatory mechanisms presents a promising therapeutic avenue to modulate immune responses, mitigate tissue damage, and improve outcomes in a range of inflammatory and autoimmune diseases [45].

## Mechanisms of NETosis

**Fig. 2:**
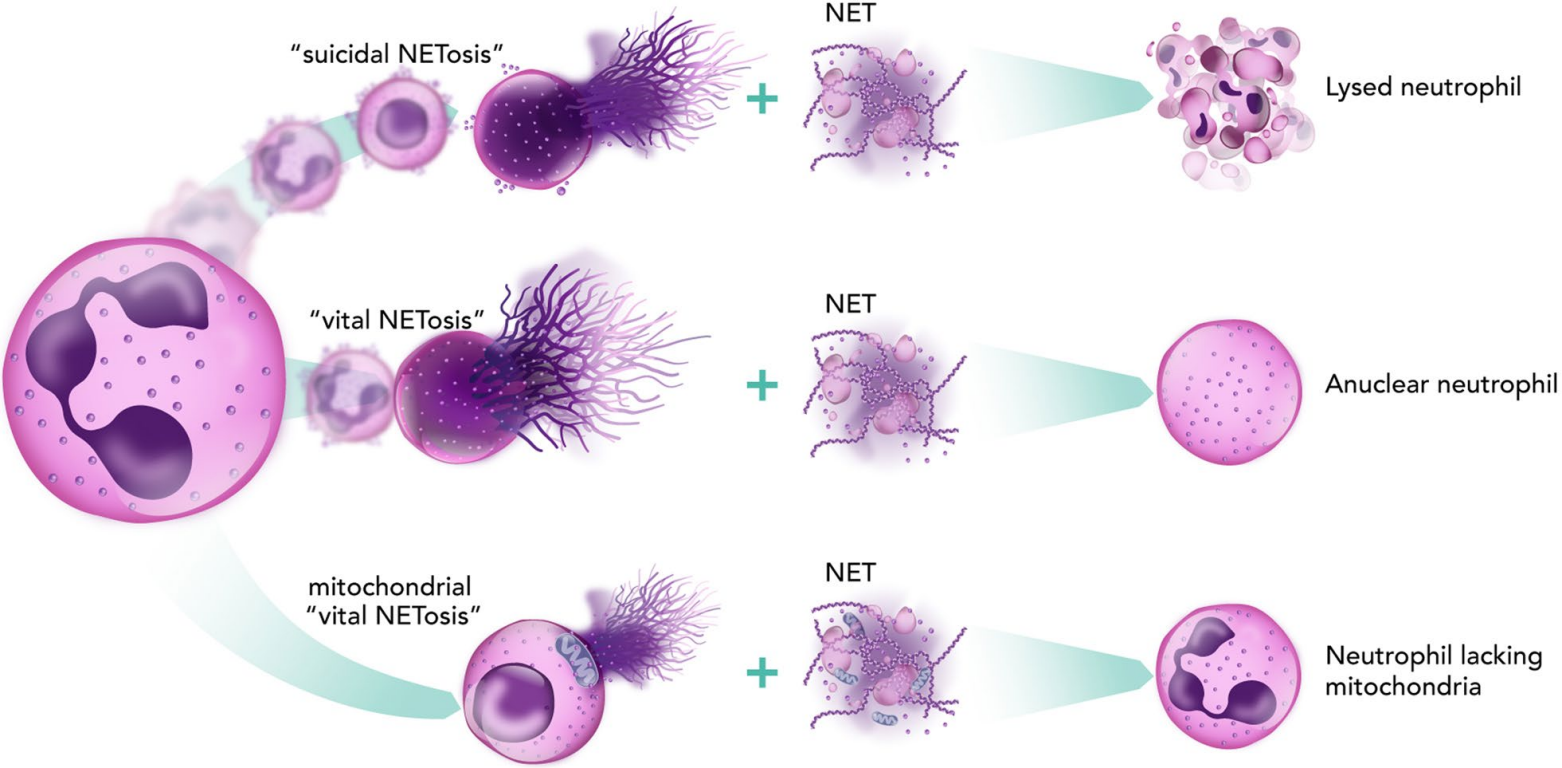
Three different types of NETosis: suicidal, vital and mitochondrial. *NET* neutrophil extracellular trap. NETosis represents a specialised form of programmed cell death wherein neutrophils release NETs, complex structures composed of chromatin fibres, histones, and antimicrobial proteins. This process, first described by Brinkmann et al. [15], has emerged as a crucial mechanism in innate immunity and inflammation. Three distinct forms of NETosis have been identified: suicidal, vital, and mitochondrial NETosis. Suicidal NETosis, the classical pathway, involves a terminal process resulting in neutrophil lysis. This mechanism is characterised by chromatin decondensation, nuclear membrane breakdown, and mixing of nuclear contents with granular antimicrobial proteins [29]. The process typically requires 2–4 h and involves the generation of reactive oxygen species (ROS) through NADPH oxidase activation. Peptidylarginine deiminase 4 (PAD4) catalyses histone citrullination, facilitating chromatin decondensation. The final stage involves plasma membrane rupture and NET release, leaving behind a lysed neutrophil [40]. Vital NETosis, in contrast, represents a rapid response mechanism occurring within 30–60 min, wherein neutrophils remain viable and retain their antimicrobial functions after NET release [187]. This process, often triggered by bacterial components or inflammatory stimuli, involves the expulsion of nuclear DNA through vesicular transport while maintaining plasma membrane integrity. The resulting anuclear neutrophils continue to perform essential functions such as phagocytosis and chemotaxis, representing an evolutionary adaptation that preserves neutrophil functionality while deploying NETs [188]. Mitochondrial NETosis represents a distinct pathway characterised by the release of mitochondrial rather than nuclear DNA. This mechanism, first reported by in 2009 [189], occurs in response to specific stimuli and results in neutrophils lacking mitochondria but maintaining nuclear integrity. The process involves selective packaging of mitochondrial DNA with antimicrobial proteins, followed by their controlled release. This form of NETosis may represent a less destructive response mechanism compared with suicidal NETosis. The figure illustrates these three distinct NETosis pathways, highlighting their unique characteristics and outcomes. The top panel shows suicidal NETosis, resulting in complete cell lysis and NET release. The middle panel depicts vital NETosis, where the neutrophil remains intact as an anuclear cell following NET release. The bottom panel illustrates mitochondrial NETosis, showing the selective release of mitochondrial DNA while maintaining cellular integrity

NETosis is induced by three distinct mechanisms: suicidal, vital and mitochondrial NETosis (Fig. 2). Suicidal NETosis takes several hours to complete compared with the rapid mechanisms of vital and mitochondrial NETosis that do not involve cell death [31, 46]. The distinction between these mechanisms has implications in sepsis. Suicidal NETosis can contribute to the depletion of neutrophils and can potentially lead to immunosuppression, a frequent problem in late-stage sepsis. Conversely, vital and mitochondrial NETosis allow neutrophils to continue their antimicrobial functions even after NET release. Notably, mitochondrial NETosis is a major source of extracellular mitochondrial DNA in the plasma of patients with sepsis; levels correlate with disease severity and are associated with poor clinical outcomes [47]. Understanding these mechanisms will be critical in developing targeted therapies for sepsis, aiming to balance the beneficial antimicrobial effects of NETs with the potential for tissue damage and inflammation.

## NETosis as a primary source of DAMPs

NETosis is a significant source of extracellular nucleosomes and histones in sepsis [15, 40]. Hypoxia, oxidative stress and direct injury can lead to cellular damage [48]. This may result in necrotic, pyroptotic, and/or apoptotic cell death, with release of intracellular contents, including DNA, nucleosomes and histones, directly into the extracellular space [49, 50]. Here they can exist freely, packaged in extracellular vesicles, or as part of NETs [15, 17, 40, 51, 52], and can by themselves act as DAMPs, exerting profound effects on the immune system and coagulation cascade [12, 14, 50, 51].

**Fig. 3:**
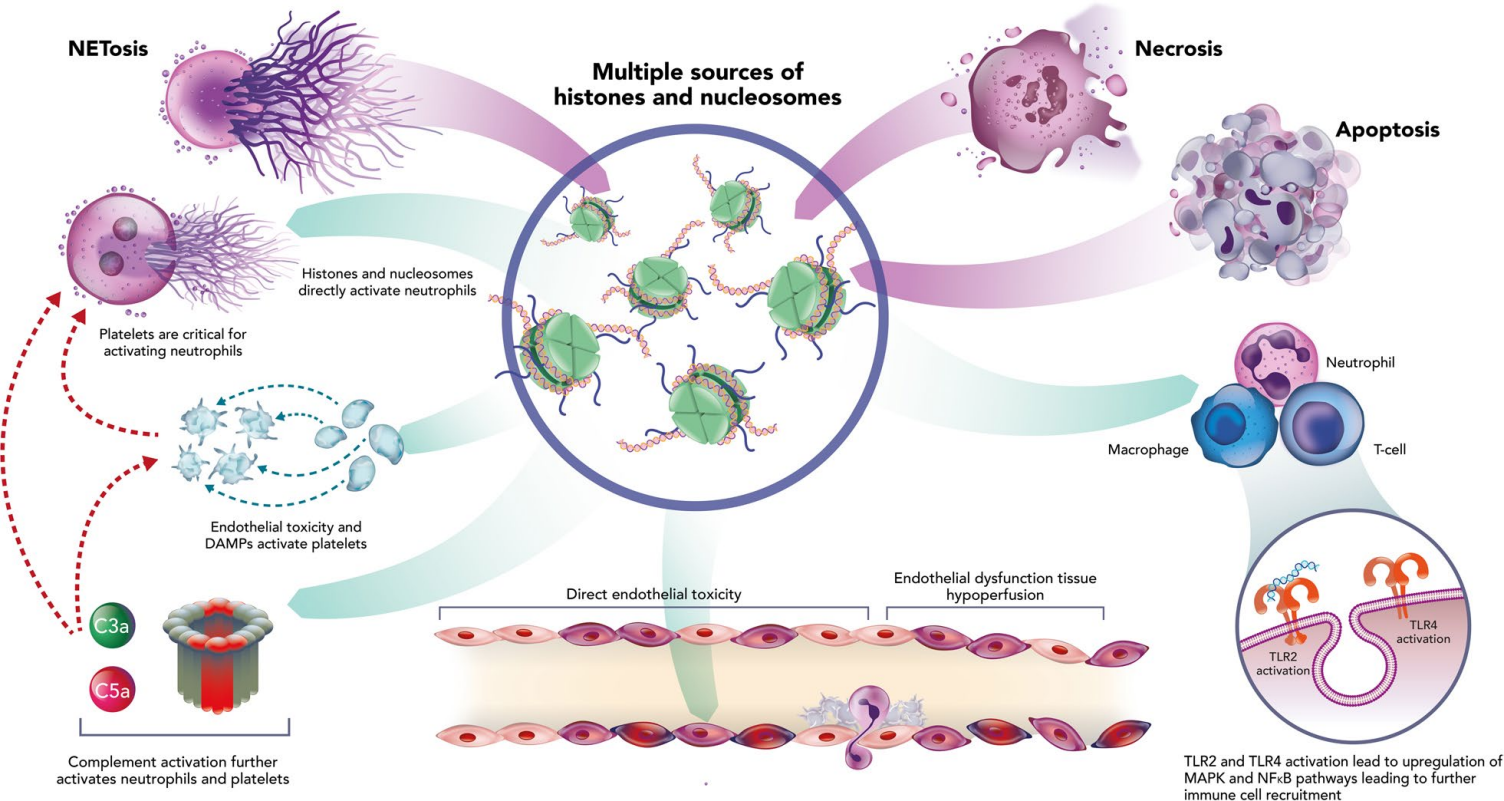
The role of histones and nucleosomes in the dysregulated host immune response. Apoptosis, necrosis and NETosis are all sources of extracellular histones. During infection NETosis is the predominant source of histones and nucleosomes (> 80%). Histones are strongly cationic and directly toxic to cell membranes. This promotes endothelial dysfunction and activation of platelets and neutrophils. Neutrophils, platelets and complement are all directly activated by histones, leading to a forward positive feedback loop. Histones can also directly activate other immune cells through interactions with TLR2 and 4, activating the pro-inflammatory mitogen-activated protein kinase (MAPK) and NLRP3 inflammasome. This figure is a reproduction and adaption from [51] under the Creative Commons Attribution 4.0 International License, (https://creativecommons.org/licenses/by/4.0/) with permission from E Silk. *DAMPs* damage-associated molecular patterns, *MAPK* mitogen-activated protein kinase, *NF-κB* nuclear factor kappa B, *NLRP3* NOD-, LRR- and pyrin domain-containing protein 3, *TLR* toll-like receptor

The overwhelming nature of sepsis may compromise the body's ability to clear apoptotic cells and cellular debris efficiently, leading to the accumulation of extracellular nucleosomes and histones. PRRs, particularly TLR2, TLR4 and TLR9, play a key role in recognising nucleosomes and histones as DAMPs [51, 53]. Extracellular histones directly bind to and activate TLR4 on immune cells, triggering nuclear factor kappa B-mediated signalling and production of pro-inflammatory cytokines, TNF-α, IL-1β and IL-6 (Fig. 3) [54, 55]. Histones may act synergistically with other TLR ligands, such as lipopolysaccharide, to enhance inflammatory responses [56]. Histones can also induce activation of the NOD-, LRR- and pyrin domain-containing protein 3 (NLRP3) inflammasome, a macromolecular protein complex and key regulator of the innate immune response [57, 58]. Canonical NLRP3 inflammasome activation occurs in two stages [59, 60]. The initial priming signal can arise from DAMP-stimulated TLR-mediated signalling, inducing the upregulation of NLRP3 and pro-inflammatory cytokines [59, 61]. DAMPs can also trigger the second ‘hit’ required for NLRP3 inflammasome assembly and activation, leading to caspase-1 stimulation and IL-1β release, by activating multiple upstream events [59, 61, 62]. Release of IL-1β further amplifies the inflammatory response in sepsis, contributing to organ dysfunction.

Excessive NET formation with subsequent release of DAMPs can lead to tissue damage, organ dysfunction and the perpetuation of the inflammatory response, contributing to the pathogenesis of inflammatory and autoimmune diseases including sepsis, acute lung injury, and SLE [51, 63]. A recent study by Malamud et al. demonstrated that myeloid inhibitory C-type lectin-like receptor (MICL/CLEC12A) regulates NET formation by directly recognising NET-associated DNA. The researchers found that MICL deficiency or inhibition leads to uncontrolled NET formation through the ROS-PAD4 pathway, creating an auto-inflammatory feedback loop [64]. Loss of MICL functionality exacerbated joint inflammation in rheumatoid arthritis models. MICL acts as a PRR for NETs on neutrophils, inhibiting further neutrophil activation and NET formation upon recognition. Of note, the authors detected similarly inhibitory anti-MICL autoantibodies in patients with lupus and severe coronavirus disease 2019 (COVID-19).

## NETs play a central role in the innate immune response and immunothrombosis

In addition to their crucial role in trapping and killing pathogens, NETs coordinate immune responses via their interactions with immune cells and mediators [40]. For example, NETs activate plasmacytoid dendritic cells by engaging TLR9 on the cell surface and stimulating production of type I interferons (IFNs). Type I IFNs are critical for activating the adaptive immune response, including antigen-specific T- and B-cell responses [65, 66]. In vitro NETs activate T cells directly by lowering their activation threshold and inducing co-stimulatory signalling [67]. They also interact with macrophages, promoting cytokine release and amplifying the immune response [68]. These interactions highlight the central role of NETs in orchestrating the complex interplay between innate and adaptive immunity [69]. Considerable crosstalk exists between neutrophils and macrophages. In sepsis, macrophage pyroptosis, a highly inflammatory form of lytic-programmed cell death regulates NET formation. Pyroptotic macrophage-derived microvesicles cause tissue damage and can activate coagulation pathways [70]. While neutrophils can endocytose pyroptotic microvesicles containing macrophage mitochondria, this induces neutrophil mitochondrial dysfunction and further NET formation via the mitochondrial ROS/Gasdermin D axis [70]. Damage to the endothelium during sepsis may also be driven by the ability of activated endothelial cells to induce NET formation and their susceptibility to NET-mediated cell death [71]. This endothelial damage is a key component of thromboinflammation, contributing to microvascular dysfunction and organ failure in sepsis [72].

Another important aspect of NET function is their role in complement activation. Complement components such as properdin, C3 and factor B have been found deposited on NETs [7, 73, 74]. Together with NET-associated granular proteins and DAMPs, these are responsible for NET-mediated complement activation [7, 73, 74]. Extracellular histones and nucleosomes induce complement activation via interactions with PRRs on the surface of immune cells [51]. Additional NET formation can be triggered by C3a and C5b, propagating the complement pathway further [7, 75]. C1q, the recognition molecule of the classical complement pathway, can bind directly to NETs. This binding is facilitated by the positively charged globular heads of C1q interacting with the negatively charged DNA backbone of NETs. C1q-opsonised NETs are more efficiently phagocytosed by macrophages, aiding in the clearance of NETs and potential pathogens trapped within them, reducing the potential for NET-mediated tissue damage [76]. The interaction between NETs and complement proteins can create a positive feedback loop, enhancing local inflammation, immune responses and procoagulant activities.

## NETs, complement and thrombosis

Through their dual role in pathogen capture and the promotion of thrombosis, NETs help to orchestrate immunothrombosis; a process that is both protective but potentially harmful in various disease states [5]. They contribute to thrombin generation through several mechanisms, including activation of the intrinsic coagulation pathway, inactivation of endogenous anticoagulants, and provision of a scaffold for the assembly of coagulation factors [77]. In addition, nucleosome and histone components also play a significant role in the dysregulated coagulation observed in sepsis.

Coagulation and thrombin formation can be promoted by the histone component of NETs, inducing platelet activation and aggregation via TLR2- and TLR4-mediated pathways [56]. Histone-induced platelet activation results in the exposure of phosphatidylserine on the platelet surface, providing a procoagulant surface [56, 78]. Increased platelet activation directly enhances and propagates thrombin generation, leading to a feed-forward loop supporting further platelet activation. Besides platelets, histones can induce surface expression of phosphatidylserine on red blood cells in vitro, supporting prothrombinase activity and shortening clotting times in plasma [79]. Histones also have the capacity to inactivate endogenous anticoagulants, such as tissue factor (TF) pathway inhibitor and thrombomodulin, promoting further thrombin generation and suppression of protein C activation [80, 81].

**Fig. 4:**
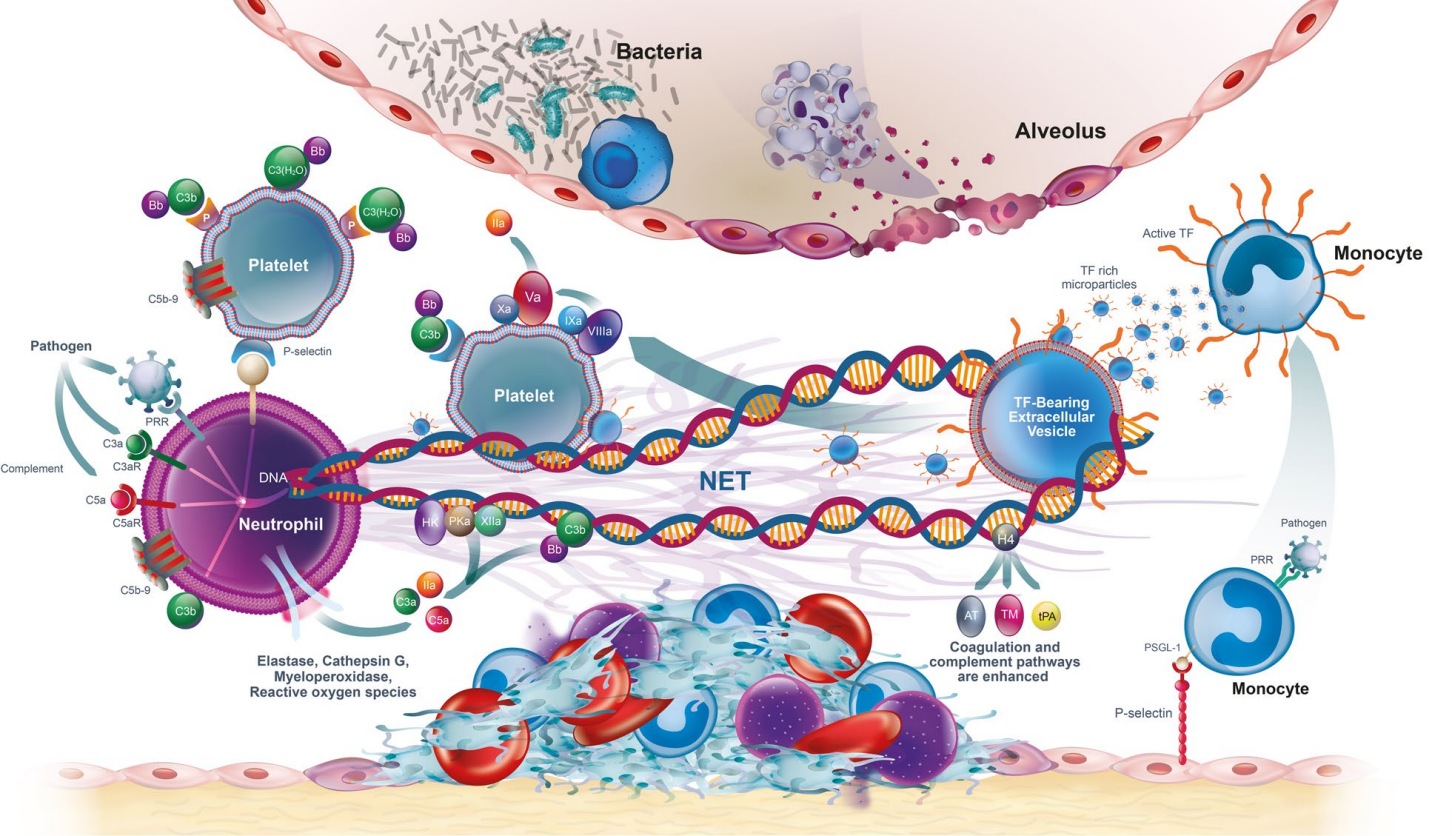
Pathogenic invasion of a pulmonary capillary, illustrating the link between thrombin generation, complement and NETs. Infection of endothelial cells disrupts the basement membrane, stimulating cell-surface expression of TF and P-selectin and, therein, the recruitment of neutrophils and platelets. Activation of complement promotes further activation of platelets and neutrophils. Platelets are activated by both the classical and alternative complement pathways. C3b can directly bind to and activate neutrophils. Neutrophils have additional receptors for C5a and C3a. Platelets can activate neutrophils directly by P-selectin glycoprotein ligand-1 (PSGL-1). NETs catch and bind platelets providing a scaffold to further support thrombin generation and the recruitment of immune cells. Monocytes detect the invading pathogen and are recruited to the site of endothelial damage/disruption. TF-bearing microvesicles released by macrophages become ensnared in the NETs. The negative charge of the NETs activates FXII, initiating the intrinsic pathway of coagulation that results in further thrombin generation. Finally, histones in NETs antagonise the action of the natural anticoagulants, thrombomodulin and activated protein C. As NETs can extend over 50 μm, with the normal diameter of a pulmonary capillary being 20–30 μm, there is a potential risk of complete obstruction of the microcirculation. This figure is a reproduction and adaption from [7] under the Creative Commons Attribution 4.0 International License, (https://creativecommons.org/licenses/by/4.0/) with permission from E.L.G Pryzdial. *AT* antithrombin, *FXII* factor XII, *HK* high molecular weight kininogen, *NET* neutrophil extracellular trap, *P* properdin, *PKa* kallikrein, *PRR* pattern recognition receptor, *P-selectin* platelet selectin, *PSGL-1* P-selectin glycoprotein ligand-1, *TF* tissue factor, *TM* thrombomodulin, *tPA* tissue-type plasminogen activator

Excessive NET release triggers thrombin generation by activating the intrinsic coagulation pathway [82] through exposure of negative charges on damaged cells or foreign surfaces [83]. This excessive NET formation and subsequent activation of coagulation cascades is a hallmark of thromboinflammation, where the protective functions of immunothrombosis become dysregulated [10]. Circulating free nucleosomes, released during excessive NETosis provide the negative charge that can directly activate factor XII (FXII) [7, 84]. Activation of FXII by nucleosomes generates FXIIa which subsequently activates FXI (Factor XI) and FIX (Factor IX). Activation of FIX leads to formation of the tenase complex (FIXa-FVIIIa) which activates FX [85]. FXa then forms a prothrombinase complex with FVa, leading to thrombin generation. The negatively charged DNA backbone of NETs can therefore bind and concentrate coagulation factors, facilitating their activation and the formation of the tenase and prothrombinase complexes (Fig. 4) [86]. This NET-mediated assembly of coagulation factors contributing to thrombosis development occurs independently of the platelet or erythrocyte surface [87].

There is evidence that nucleosomes, NET-associated histones and free histones can initiate and propagate the extrinsic coagulation pathway. Extracellular histones can disrupt the integrity of the endothelial barrier, exposing the procoagulant subendothelial matrix to circulating factors [84, 88]. They can also induce endothelial expression of TF via TLR4- and TLR2-mediated signalling, leading to autoactivation of FVII-activating protease [89, 90]. Histone-induced endothelial activation also promotes the release of von Willebrand factor from endothelial cells, enhancing platelet adhesion and aggregation [91]. The involvement of NETs and associated DAMPs in both intrinsic and extrinsic coagulation pathways exemplifies how NETs serve as a critical link between innate immunity and coagulation in immunothrombosis [84, 86].

NETosis has been implicated in coagulopathies associated with conditions such as sepsis, COVID-19 and autoimmune disorders (SLE, antiphospholipid syndrome [APS]) [6, 41, 92]. These conditions represent states of excessive thromboinflammation [93]. In sepsis, excessive NET formation has been associated with the development of sepsis-induced coagulopathy and multiple organ failure [94, 95]. NETs contributed to the hypercoagulable state in patients with COVID-19, potentially explaining the high incidence of thrombotic complications in severe cases [96]. In SLE and APS, NETs are implicated in the development of thrombosis and cardiovascular disease in affected individuals [97]. Their role in cardiovascular disease has been reviewed in detail by Stark and Massberg [8]. The most extreme form of systemic coagulopathy, disseminated intravascular coagulation, is associated with severe bacterial infections [9, 98, 99]. Sepsis-induced coagulopathy develops in about 35% of sepsis cases [9]. Understanding the role of NETs in these pathological states of thromboinflammation is crucial for developing targeted therapies to modulate the immune response and improve patient outcomes [100].

The complement system and NETs engage in bidirectional interactions that can amplify inflammatory responses following trauma. Complement activation, particularly through C5a-C5aR signalling, triggers NET formation via NADPH oxidase-dependent ROS generation and peptidylarginine deiminase 4 (PAD4)-mediated histone citrullination [15, 87]. In turn, NETs activate complement through multiple mechanisms: extruded DNA and histones serve as platforms for C1q binding to initiate the classical pathway, while NET-associated proteases can directly cleave C3 and C5 to generate anaphylatoxins [74]. This creates a potentially harmful positive feedback loop in trauma patients, as NET-induced complement activation recruits additional neutrophils and promotes further NET formation, potentially contributing to organ dysfunction. Therapeutic strategies targeting this axis show promise—C5a inhibition reduces NET formation in experimental models, while DNase treatment both degrades NETs and limits complement activation [101, 102]. While this complement-NET interaction represents a key mechanism linking innate immunity and inflammation in trauma, further research is needed to fully characterise the spatial and temporal dynamics of these interactions and optimise therapeutic approaches.

## Some microorganisms subvert the antimicrobial properties of NETs

While NETs play a crucial role in the innate immune response against invading microorganisms, some pathogens have evolved strategies that can manipulate NETs to enhance their survival and increase pathogenicity (Table 1) [4, 103–117]. These strategies include evading NET capture, resisting NET-mediated killing, degrading NETs, and exploiting NETs for enhanced virulence [111, 118–120]. To evade NET capture some pathogens have evolved surface modifications that reduce NET binding, allowing them to escape entrapment and destruction [111]. For example, *Streptococcus pneumoniae* has a negatively charged polysaccharide capsule that creates an electrostatic repulsion with the negatively charged DNA backbone of NETs, thereby preventing entrapment [112, 121]. Similarly, *Klebsiella pneumoniae* produces a capsule that inhibits NET formation and reduces bacterial capture [122]. Other bacteria modify their surface proteins to evade NET capture [103]. Microorganisms can also work cooperatively, forming biofilms that act as physical barriers, preventing neutrophil infiltration and NET formation [118, 120, 123]. Others have developed mechanisms to resist the antimicrobial effects of NETs, enabling them to survive and propagate within these structures [124]. Many bacteria produce nuclease enzymes or hijack host DNases to degrade and destroy the DNA backbone of NETs [104, 105, 112, 120, 125]. These include *Streptococcus pyogenes* which secretes DNase Sda1 and *Staphylococcus aureus* which induces release of host DNases [106, 120]. In contrast, LasR-deficient *Pseudomonas aeruginosa*, *Yersinia pestis*, and *Bordetella pertussis* strains reduce or prevent NET formation [116, 126, 127]. In *Mycobacterium tuberculosis* infections, NET formation in tuberculosis lesions may contribute to tissue damage and exacerbate inflammation, potentially facilitating bacterial dissemination and disease progression [127]*.* The mechanisms employed by microorganisms to manipulate NETs and their consequences for pathogenicity and survival are summarised in Table 1.

**Table 1:** Mechanisms employed by microorganisms to manipulate NETs and their consequences for pathogenicity and survival

Microorganism	Mechanism of NET manipulation	Consequence for pathogenicity and survival
*S. aureus*	• Secretion of Nuc and AdsA enzymes degrades surrounding NET DNA, reducing entrapment and generating deoxyadenosine [104, 107] • Deoxyadenosine triggers macrophage apoptosis and disrupts efferocytosis of apoptotic neutrophils and NETs [107] • *S. aureus* biofilms secrete leukocidins, Panton-Valentine leucocidin (PVL) and HlgAB to promote and inhibit NETosis allowing them to evade capture [108]	Facilitates persistence of infections [104, 107]
*S. agalactiae* (Group B Streptococcus, GBS)	• Some species of *S. agalactiae* secrete the nuclease, NucA, to degrade the DNA NET matrix enabling them to evade NET entrapment [105]	Promotes virulence and persisting infection [105]
*S. pyogenes* (Group A Streptococcus, GAS)	• Surface M protein binds fibrinogen forming a protective coat around the bacteria [103] • Secretion of DNase Sda1 facilitates bacterial survival and escape from NETs by degrading the DNA matrix [106] • DNase Sda1 activity may exert a selective pressure that increases virulence, shifting the infection from local to systemic [109]	Promotes bacterial survival and dissemination, increasing pathogenicity and facilitating tissue infiltration [106, 109]
*N. meningitidis* (meningococcus, Nm)	• Modification of lipid A of meningococcal lipopolysaccharide (LPS) with PEA protects the organism from bactericidal activity of NET-bound cathepsin G [110] • Expression of the high-affinity zinc uptake receptor ZnuD protects meningococci from NET-mediated nutritional immunity [110] • Spontaneously released outer membrane vesicles inhibit *N.* *meningitidis* binding to NETs by saturating meningococcal binding sites, neutralising NET action [110]	Promotes bacterial survival, dissemination and rapid progression of meningococcal disease [110]
*S. pneumoniae* (Pneumococcus)	*S. pneumoniae* has evolved several strategies to evade and exploit NETs Capsule protects against NET trapping: • The polysaccharide capsule of *S. pneumoniae* reduces bacterial trapping in NETs by 4–12 fold compared to non-encapsulated strains [111]. This allows encapsulated pneumococci to avoid confinement by NETS Resistance to NET-mediated killing: • Unlike many pathogens, encapsulated *S. pneumoniae* is not killed by antimicrobial components in NETs [112, 113]. This allows trapped bacteria to survive Endonuclease degrades NETs: • *S. pneumoniae* expresses a surface endonuclease called EndA that degrades the DNA scaffold of NETs, allowing bacteria to escape [112]. EndA-deficient mutants remain trapped in NETs and show reduced virulence in mouse models D-alanylation of lipoteichoic acids: • Modification of lipoteichoic acids with D-alanine introduces positive charge to the bacterial surface, reducing susceptibility to cationic antimicrobial peptides in NETs [111]. This promotes resistance to NET killing Pneumolysin activates NET formation: • The pneumococcal toxin pneumolysin induces NET formation [114]. While this may seem counterproductive, it may benefit the bacteria by depleting neutrophils Biofilm formation: • Pneumolysin promotes biofilm formation, which may help pneumococci evade capture by NETs [4]	Escaping NETs promotes spread of *Pneumococci* from the upper respiratory tract to the lung, and from lung to bloodstream during pneumonia
*Y. pestis*	• Platelets intoxicated by *Y. pestis* induced Type 3 secretion system expression, have a diminished response to prothrombotic stimuli. The T3SS allows *Y. pestis *to escape entrapment in platelet-neutrophil thrombi. Spinner et al. show that *Y. pestis* with Type 2 secretion system grown at 37°C inhibits neutrophil production of ROS, attenuating NET formation and reducing exposure to the antimicrobial effects of NETs [115, 116]	Facilitates bacterial survival and dissemination of infection by enabling *Y.* *pestis* to reduce NETosis in platelet thrombi [115, 116]
*B. pertussis*	• Secretion of adenylate cyclase toxin by *B. pertussis* generates supraphysiological levels of intracellular cAMP (Cyclic AMP), preventing the oxidative burst required for NETosis [117] • Adenylate cyclase toxin impairs neutrophil apoptosis and neutrophil-mediated phagocytosis [117]	Facilitates bacterial survival and dissemination; potentially influences local tissue damage during infection [117]
*LasR-deficient Pseudomonas*	• *LasR-deficient* strains alter both the quantity and quality of NETs, reducing overall NET formation while changing the spatial distribution of key antimicrobial proteins like BPI and neutrophil elastase on NET structures [126] • The reduction in NET formation occurs through LasR-regulated virulence factors (elastase LasB and protease LasA)	Up to 63% of chronically infected cystic fibrosis patients have *P. aeruginosa* with inactivating mutations in lasR, suggesting selective pressure for this adaptation [126] The ability to reduce NET formation represents a specific bacterial immune evasion strategy that may contribute to persistent infection in these patients

*ACT* adenylate cyclase toxin, *B. pertussis Bordetella pertussis*, *cAMP* cyclic AMP, *HlgAB* γ-haemolysin AB, *LPS* lipopolysaccharide, *N. meningitidis Neisseria meningitidis*, *NET* neutrophil extracellular trap, *PVL* Panton-Valentine leucocidin, *PEA* phosphoethanolamine, *ROS* reactive oxygen species, *S. agalactiae Streptococcus agalactaie*, *S. aureus Staphylococcus aureus*, *S. pneumoniae Streptococcus pneumoniae*, *S. pyogenes Streptococcus pyogenes*, *T3SS* type III secretion system, *Y. pestis Yersinia pestis*

## Future strategies to manipulate NETs and improve patient outcomes

The pathological impact of excessive or dysregulated NET formation offers potential therapeutic targets for a range of conditions such as sepsis, thrombosis and autoimmune disorders. Recognising that NETs and associated DAMPs are critical mediators of inflammation and coagulopathy in sepsis has opened new avenues for therapeutic interventions. Key therapeutic strategies include inhibition of NET formation, promotion of specific NET degradation and targeting specific NET components (Table 2) [128–146].

**Table 2:** Potential anti-NET therapeutics

Therapeutic strategy	Agent or device	Description	References
Inhibition of NET formation	Cl-amidine	Small-molecule PAD4 inhibitor	[129–131]
	GSK484	Reversible, selective PAD4 inhibitor	[132]
	DPI	Small-molecule, non-specific NOX inhibitor	[133]
	GSK2795039	Small-molecule NOX 2 inhibitor	[134]
	APX-115	Specific NOX inhibitor	[135, 136]
Promotion of NET degradation	Dornase alfa (rhDNase I)	NET DNA	[137]
	C1q	DNase 1	[138]
Targeting NET components	AZD3241	Small-molecule selective and irreversible MPO inhibitor	[140]
	Sivelestat	Small-molecule NE inhibitor	[128, 141–143]
	3D2D-APC, 3D2D2A-APC	Rationally designed recombinant protein variants of APC	[174]
	M6229	Low-anticoagulant fraction of unfractionated heparin (UFH)	[139]
	NucleoCapture™	Therapeutic apheresis device that selectively removes NETs from blood	[144–146]

*APC* activated protein C, *DPI* diphenyleneiodonium, *MPO* myeloperoxidase, *NADPH* nicotinamide adenine dinucleotide phosphate, *NE* neutrophil elastase, *NET* neutrophil extracellular trap, *NOX* NADPH oxidase, *PAD4* peptidylarginine deiminase 4, *rhDNase I* recombinant human DNase I, *UFH* unfractionated heparin

### Inhibition of NET formation

NET formation can be inhibited by targeting molecular pathways underlying NETosis, but whether partial or full inhibition of NET formation should be targeted still needs to be considered. Potential inhibitors of NET formation include small molecules, peptides and antibodies. PAD4 is a key enzyme in the NETosis pathway that catalyses histone citrullination leading to chromatin decondensation and NET formation [128]. Inhibition of PAD4 reduced NET formation both in vitro and in vivo [147]. Cl-amidine, a small-molecule PAD4 inhibitor, attenuated NET formation and improved outcomes in animal models of sepsis, thrombosis and autoimmune diseases [129–131]. GSK484, another PAD4 inhibitor, was well tolerated in a phase 1 clinical trial in healthy volunteers [132]. However, the efficacy of PAD4 inhibitors in human disease remains to be determined. Long-term inhibition of PAD4 may also have unintended consequences on other cellular processes, such as gene regulation and cell differentiation [148].

NOX, a key enzyme in the generation of ROS, is essential for NET formation [29]. Inhibition of NOX reduced NET formation both in vitro and in vivo [149]. Diphenylene iodonium (DPI), a non-specific NOX inhibitor, inhibited NET formation in models of sepsis and lupus nephritis. However, DPI has off-target effects and can inhibit other flavoenzymes, limiting its therapeutic potential [133]. More specific NOX inhibitors, such as GSK2795039 and APX-115, show promise in preclinical studies [134–136]. Nevertheless, the potential risks of NOX inhibition should be considered, as ROS play important roles in host defence and cell signalling [150].

### Promotion of NET degradation

Another therapeutic strategy is to promote NET degradation, either by enhancing activity of endogenous DNases or by administering such enzymes exogenously. Recombinant human DNase I (rhDNase I) degrades NETs [151]. Improved outcomes were seen in animal models of sepsis, acute lung injury and autoimmune diseases following administration of rhDNase I [151–153]. In humans, rhDNase I (dornase alfa) is approved for the treatment of cystic fibrosis where it reduces sputum viscoelasticity and improves lung function [137, 154, 155]. rhDNase I showed preclinical effectiveness against severe acute respiratory syndrome coronavirus 2 infection [151]. However, the efficacy of this therapy in other conditions associated with excessive NET formation remains to be established. Recent research has highlighted DNase's potential in treating thrombotic conditions, where NETs play a crucial role in promoting thrombosis [40]. Studies have demonstrated that NETs provide a scaffold for platelet adhesion and activation, contributing to thrombus formation. DNase has shown promise in treating thrombosis by degrading NET structures that promote blood clot formation. Clinical studies found DNase treatment reduced thrombus size and improved outcomes in conditions like deep vein thrombosis [156]. In experimental models, recombinant human DNase prevented thrombosis and reduced mortality [157, 158].

The therapeutic potential of DNase extends beyond its direct NET-degrading properties. Research suggests that DNase treatment may modulate inflammatory responses by reducing the availability of NET-associated DAMPs [159]. This reduction in inflammatory stimuli could contribute to breaking the cycle of chronic inflammation observed in many NET-associated pathologies. However, challenges remain in optimising DNase therapy [160]. The timing of administration, delivery methods, and potential combination with other therapeutic agents require further investigation. Potential risks of rhDNase I treatment, such as the development of anti-DNase antibodies and the impairment of host defence, need to be considered.

Endogenous DNases, such as DNase1 and DNase1-like 3, play a crucial role in NET degradation [161]. Deficiency or inhibition of these DNases is associated with accumulation of NETs and development of autoimmune diseases such as SLE [162]. Enhancing activity of endogenous DNases may represent another therapeutic strategy to promote NET degradation. C1q, a component of the complement system, enhances DNase1 activity and facilitates NET degradation [76]. Administration of C1q reduced NET accumulation and attenuated disease severity in animal models of SLE [138]. However, the therapeutic potential of C1q in human diseases remains to be investigated and the potential risks of modulating the complement system should be considered [163].

DNase has been administered through several routes across different clinical contexts. The most established approach is inhaled or nebulised DNase (dornase alfa/Pulmozyme), which is primarily used in respiratory conditions like cystic fibrosis, bronchiolitis and COVID-19, delivered via nebuliser directly to airways without systemic absorption [164, 165]. In experimental settings, particularly in mouse models, DNase has been administered through intraperitoneal (IP) injection demonstrating that DNase at doses around 20 mg/kg could be effective, with timing being a crucial factor; administration at 4 or 6 h post-infection showed better outcomes than immediate treatment [152]. Intratracheal (IT) administration has also been studied in experimental models, with direct instillation into the trachea. Lefrançais et al. (2018) used this method in their research, administering doses of 2,000–4,000 units at regular intervals of 8–10 h [155]. The timing of administration appears crucial to therapeutic success, with evidence suggesting that delayed administration (4–6 h after infection) may be more beneficial than immediate treatment in some contexts [152]. While human trials have primarily focused on inhaled DNase, intravenous administration remains largely experimental, requiring further research to establish optimal protocols and safety parameters.

### Targeting NET components

As described above, NETs comprise many components, including nucleosomes, histones, MPO and NE, which contribute to NET-mediated pathology [166]. Targeting these components may also be potentially advantageous, for example, histones exert cytotoxic and pro-inflammatory effects [53]. Neutralisation of histones with antibodies or small molecules (e.g., M6229, a low-anticoagulant fraction of unfractionated heparin) attenuated NET-mediated tissue damage and improved outcomes in animal models of sepsis, trauma and autoimmune diseases [139, 167]. A phase 1 study (NCT05208112) of M6229 in critically ill adults with sepsis has recently been completed [168]. The potential risks of modulating histone functions, such as impaired host defence and altered gene regulation, should be considered. MPO, a key enzyme in the generation of hypochlorous acid, is a potent oxidant [169], contributing to the antimicrobial and pro-inflammatory effects of NETs [170]. Inhibition of MPO reduced NET formation and attenuated NET-mediated tissue damage both in vitro and in vivo [171]. AZD3241, a small-molecule MPO inhibitor, has been evaluated in clinical trials for the treatment of neurodegenerative brain disorders [140]. However, their efficacy in NET-associated pathologies remains to be investigated and the potential risks of MPO inhibition, such as increased susceptibility to infection, should be considered [37]. Activated protein C (APC) can cleave histone H3, reducing their cytotoxicity [172]. However, due to the anticoagulant properties of APC and increased risk of bleeding, recombinant APC is no longer used therapeutically [173]. Two APC variants (3D2D-APC and 3D2D2A-APC) were designed with reduced anticoagulant activity and shown to have increased binding for H3 and proteolytic activity, reducing its cytotoxic effects on endothelial cells [174]. NE, a serine protease that contributes to the proteolytic activity of NETs, has been implicated in the pathogenesis of various inflammatory and autoimmune diseases [33]. Sivelestat, a small-molecule NE inhibitor, reduced NET formation and attenuated NET-mediated tissue damage both in vitro and in vivo [128, 141]. Sivelestat has been investigated clinically for treatment of acute lung injury and ARDS [142, 143] but its efficacy and potential risks, such as impaired host defence, in other NET-associated diseases remains to be determined [175]. The administration of DNase may reduce circulating nucleosome levels, and as discussed previously, has been shown to degrade NETs [151] and reduce organ damage in animal models of sepsis [152]. An alternative to a pharmacological approach is extracorporeal removal of NETs. NucleoCapture™ therapeutic plasmapheresis utilises histone H1.3 protein as a selective DNA adsorber to remove NETs from blood. It improved organ function and survival in a porcine model of sepsis [144, 145] and has been given to patients with sepsis in a pilot study (NCT04749238) [146]. The potential anti-NET therapeutics are summarised in Table 2.

## Biomarkers of NETs

Prompt diagnosis and treatment are crucial for ensuring the best outcomes for patients with sepsis. As NETs are an important part of the immune response, care needs to be taken when selecting treatments, as blocking or disrupting low levels of NETs could, as with any immunomodulatory agent, have the potential for increased susceptibility to opportunistic infections. The ‘current’ recommended ‘gold standard’ for NET visualisation remains fluorescence microscopy utilising DNA stains combined with immunofluorescence staining for specific markers, including histone H3, neutrophil elastase, and myeloperoxidase [40]. The microscopic analysis should be complemented by cell-free DNA quantification and nucleosome detection [176]. The accurate, reproducible, quantifiable and translational diagnosis of NETosis is being worked on by the International Society of Thrombosis and Haemostasis. This is a critical step in informing and enabling the standardisation and comparison of further studies.

A range of tools can be used to detect and quantify NET formation and the most common techniques have been reviewed in detail by Stoimenou et al. [177]. These include immunoassays such as enzyme-linked immunosorbent assay (ELISA) and Western blot, flow cytometry-based techniques and microscopy methods. ELISA is the most used, objective and quantitative method for monitoring NETs, due to its low cost and simplicity, although standardisation remains a challenge [177–179]. These tools are used in combination with NETs biomarkers such as, histones, NE, MPO, cell-free DNA, nucleosomes, and their complexes [178, 180]. However, discussion remains about the most suitable NET marker to use as a diagnostic, risk stratifier or prognostic tool. Several validated commercial ELISA tests and in-house protocols are currently available for detecting NETs, using serum or plasma levels of MPO-DNA complex, histones, and nucleosomes as surrogate markers [177–179, 181, 182]. Clinical data suggest that these circulating biomarkers may be associated with the presence and severity of thrombosis and sepsis, and correlate with hypercoagulability, mortality and organ damage [178, 180, 183, 184]. There is currently no consensus regarding biomarker thresholds for quantifying the presence of NETs in sepsis. Larger clinical trials are needed to confirm the utility of NETs biomarkers in clinical practice and specifically to guide interventions targeting NETs.

## Conclusion

NETs and associated DAMPs are crucial players in immunothrombosis, a physiological process that links innate immunity with thrombosis to contain and eliminate pathogens [5, 6, 8]. When dysregulated, this process can lead to thromboinflammation, contributing to the pathogenesis of sepsis and other inflammatory conditions [5]. Excessive release or inadequate removal of NETs can lead to the development and progression of sepsis [10]. Therefore, manipulating NETs represents a promising therapeutic strategy for sepsis and other conditions associated with excessive or dysregulated NET formation. Importantly, modulating NET formation and function may also help balance the beneficial aspects of immunothrombosis with the detrimental effects of thromboinflammation [185]. Potential approaches include inhibiting NET formation, promoting NET degradation and targeting NET components. While these approaches have shown promise in preclinical studies, their clinical efficacy and safety need to be established in humans.

Recent research has focused on targeting specific pathways involved in immunothrombosis and thromboinflammation, such as the interaction between NETs and the complement system [186]. The development of NET-targeting therapies faces several challenges such as the heterogeneity of NETs, potential off-target effects of inhibitors, and the risk of impairing host defences. Therefore, a deeper understanding of the molecular mechanisms underlying NET formation and regulation, as well as the identification of specific targets and biomarkers, will be crucial for successful translation of NET-targeting therapies into clinical practice. Moreover, the potential risks and benefits of each approach should be carefully evaluated and guided by specific biomarkers of disease severity. Future research should aim to develop therapies that can selectively modulate NET function in thromboinflammation, potentially leading to more effective treatments for sepsis and related disorders.

## Data Availability

No datasets were generated or analysed during the current study.

## Abbreviations

ACT: Adenylate cyclase toxin
APC: Activated protein C
ARDS: Acute respiratory distress syndrome
APS: Antiphospholipid syndrome
cAMP: Cyclic AMP
DAMP: Damage-associated molecular pattern
DPI: Diphenylene iodonium
ELISA: Enzyme-linked immunosorbent assay
FIX: Factor IX
FXII: Factor XII
GAS: Group A Streptococcus
GBS: Group B Streptococcus
HK: High molecular weight kininogen
IFN: Interferon
IL: Interleukin
LPS: Lipopolysaccharide
MAPK: Mitogen-activated protein kinase
MPO: Myeloperoxidase
NADPH: Nicotinamide adenine dinucleotide phosphate
NE: Neutrophil elastase
NET: Neutrophil extracellular trap
NF-κB: Nuclear factor kappa B
PAD4: Peptidylarginine deiminase 4
PRR: Pattern recognition receptor
PSGL-1: P-selectin glycoprotein ligand-1
PVL: Panton-Valentine leucocidin
rhDNase I: Recombinant human DNase I
ROS: Reactive oxygen species
SLE: Systemic lupus erythematosus
TF: Tissue factor
TLR: Toll-like receptor
TM: Thrombomodulin
TNFα: Tumour necrosis factor alpha
tPA: Tissue-type plasminogen activator
UFH: Unfractionated heparin

## Declarations

### Availability of data and materials

Not applicable, as this is a narrative review article. All data referenced is from previously published studies cited in the references section and available through their respective publishers.

## References

[1] Singer M, Deutschman CS, Seymour CW, et al. The third international consensus definitions for sepsis and septic shock (Sepsis-3). JAMA. 2016;315:801. 10.1001/jama.2016.0287. 26903338 10.1001/jama.2016.0287 PMC4968574

[2] Rudd KE, Johnson SC, Agesa KM, et al. Global, regional, and national sepsis incidence and mortality, 1990–2017: analysis for the Global Burden of Disease Study. Lancet. 2020;395:200–11. 10.1016/S0140-6736(19)32989-7. 31954465 10.1016/S0140-6736(19)32989-7 PMC6970225

[3] Fleischmann C, Scherag A, Adhikari NKJ, et al. Assessment of global incidence and mortality of hospital-treated sepsis. Current estimates and limitations. Am J Respir Crit Care Med. 2016;193:259–72. 10.1164/rccm.201504-0781OC. 26414292 10.1164/rccm.201504-0781OC

[4] Ayres JS, Schneider DS. Tolerance of infections. Annu Rev Immunol. 2012;30:271–94. 10.1146/annurev-immunol-020711-075030. 22224770 10.1146/annurev-immunol-020711-075030

[5] Engelmann B, Massberg S. Thrombosis as an intravascular effector of innate immunity. Nat Rev Immunol. 2013;13:34–45. 10.1038/nri3345. 23222502 10.1038/nri3345

[6] Bonaventura A, Vecchié A, Dagna L, et al. Endothelial dysfunction and immunothrombosis as key pathogenic mechanisms in COVID-19. Nat Rev Immunol. 2021;21:319–29. 10.1038/s41577-021-00536-9. 33824483 10.1038/s41577-021-00536-9 PMC8023349

[7] Pryzdial ELG, Leatherdale A, Conway EM. Coagulation and complement: key innate defense participants in a seamless web. Front Immunol. 2022;13: 918775. 10.3389/fimmu.2022.918775. 36016942 10.3389/fimmu.2022.918775 PMC9398469

[8] Stark K, Massberg S. Interplay between inflammation and thrombosis in cardiovascular pathology. Nat Rev Cardiol. 2021;18:666–82. 10.1038/s41569-021-00552-1. 33958774 10.1038/s41569-021-00552-1 PMC8100938

[9] Levi M, Scully M. How I treat disseminated intravascular coagulation. Blood. 2018;131:845–54. 10.1182/blood-2017-10-804096. 29255070 10.1182/blood-2017-10-804096

[10] Jackson SP, Darbousset R, Schoenwaelder SM. Thromboinflammation: challenges of therapeutically targeting coagulation and other host defense mechanisms. Blood. 2019;133:906–18. 10.1182/blood-2018-11-882993. 30642917 10.1182/blood-2018-11-882993

[11] Wilhelm G, Mertowska P, Mertowski S, et al. The crossroads of the coagulation system and the immune system: interactions and connections. Int J Mol Sci. 2023;24:12563. 10.3390/ijms241612563. 37628744 10.3390/ijms241612563 PMC10454528

[12] Krysko DV, Agostinis P, Krysko O, et al. Emerging role of damage-associated molecular patterns derived from mitochondria in inflammation. Trends Immunol. 2011;32:157–64. 10.1016/j.it.2011.01.005. 21334975 10.1016/j.it.2011.01.005

[13] Takeuchi O, Akira S. Pattern recognition receptors and inflammation. Cell. 2010;140:805–20. 10.1016/j.cell.2010.01.022. 20303872 10.1016/j.cell.2010.01.022

[14] Chen GY, Nuñez G. Sterile inflammation: sensing and reacting to damage. Nat Rev Immunol. 2010;10:826–37. 10.1038/nri2873. 21088683 10.1038/nri2873 PMC3114424

[15] Brinkmann V, Reichard U, Goosmann C, et al. Neutrophil extracellular traps kill bacteria. Science. 2004;303:1532–5. 10.1126/science.1092385. 15001782 10.1126/science.1092385

[16] Czaikoski PG, Mota JMSC, Nascimento DC, et al. Neutrophil extracellular traps induce organ damage during experimental and clinical sepsis. PLoS ONE. 2016;11: e0148142. 10.1371/journal.pone.0148142. 26849138 10.1371/journal.pone.0148142 PMC4743982

[17] Xu J, Zhang X, Pelayo R, et al. Extracellular histones are major mediators of death in sepsis. Nat Med. 2009;15:1318–21. 10.1038/nm.2053. 19855397 10.1038/nm.2053 PMC2783754

[18] van der Poll T, van de Veerdonk FL, Scicluna BP, Netea MG. The immunopathology of sepsis and potential therapeutic targets. Nat Rev Immunol. 2017;17:407–20. 10.1038/nri.2017.36. 28436424 10.1038/nri.2017.36

[19] Krémer V, Godon O, Bruhns P, et al. Isolation methods determine human neutrophil responses after stimulation. Front Immunol. 2023;14:1301183. 10.3389/fimmu.2023.1301183. 38077317 10.3389/fimmu.2023.1301183 PMC10704165

[20] Kolaczkowska E, Kubes P. Neutrophil recruitment and function in health and inflammation. Nat Rev Immunol. 2013;13:159–75. 10.1038/nri3399. 23435331 10.1038/nri3399

[21] Blanter M, Cambier S, De Bondt M, et al. Method matters: effect of purification technology on neutrophil phenotype and function. Front Immunol. 2022;13: 820058. 10.3389/fimmu.2022.820058. 35222394 10.3389/fimmu.2022.820058 PMC8866851

[22] Rosales C. Neutrophil: a cell with many roles in inflammation or several cell types? Front Physiol. 2018;9:113. 10.3389/fphys.2018.00113. 29515456 10.3389/fphys.2018.00113 PMC5826082

[23] Nguyen GT, Green ER, Mecsas J. Neutrophils to the ROScue: mechanisms of NADPH oxidase activation and bacterial resistance. Front Cell Infect Microbiol. 2017;7:373. 10.3389/fcimb.2017.00373. 28890882 10.3389/fcimb.2017.00373 PMC5574878

[24] Zukas K, Cayford J, Serneo F, et al. Rapid high-throughput method for investigating physiological regulation of neutrophil extracellular trap formation. J Thromb Haemost. 2024;22:2543–54. 10.1016/j.jtha.2024.05.028. 38866247 10.1016/j.jtha.2024.05.028

[25] Ng LG, Ostuni R, Hidalgo A. Heterogeneity of neutrophils. Nat Rev Immunol. 2019;19:255–65. 10.1038/s41577-019-0141-8. 30816340 10.1038/s41577-019-0141-8

[26] Mukhopadhyay M. Unraveling immune cell behavior. Nat Methods. 2022;19:272–272. 10.1038/s41592-022-01435-0. 35277702 10.1038/s41592-022-01435-0

[27] Mestas J, Hughes CCW. Of mice and not men: differences between mouse and human immunology. J Immunol. 2004;172:2731–8. 10.4049/jimmunol.172.5.2731. 14978070 10.4049/jimmunol.172.5.2731

[28] Nauseef WM, Borregaard N. Neutrophils at work. Nat Immunol. 2014;15:602–11. 10.1038/ni.2921. 24940954 10.1038/ni.2921

[29] Fuchs TA, Abed U, Goosmann C, et al. Novel cell death program leads to neutrophil extracellular traps. J Cell Biol. 2007;176:231–41. 10.1083/jcb.200606027. 17210947 10.1083/jcb.200606027 PMC2063942

[30] Kaplan MJ, Radic M. Neutrophil extracellular traps: double-edged swords of innate immunity. J Immunol. 2012;189:2689–95. 10.4049/jimmunol.1201719. 22956760 10.4049/jimmunol.1201719 PMC3439169

[31] Delgado-Rizo V, Martínez-Guzmán MA, Iñiguez-Gutierrez L, et al. Neutrophil extracellular traps and its implications in inflammation: an overview. Front Immunol. 2017;8:81. 10.3389/fimmu.2017.00081. 28220120 10.3389/fimmu.2017.00081 PMC5292617

[32] Wang Y, Li M, Stadler S, et al. Histone hypercitrullination mediates chromatin decondensation and neutrophil extracellular trap formation. J Cell Biol. 2009;184:205–13. 10.1083/jcb.200806072. 19153223 10.1083/jcb.200806072 PMC2654299

[33] Korkmaz B, Horwitz MS, Jenne DE, Gauthier F. Neutrophil elastase, proteinase 3, and cathepsin G as therapeutic targets in human diseases. Pharmacol Rev. 2010;62:726–59. 10.1124/pr.110.002733. 21079042 10.1124/pr.110.002733 PMC2993259

[34] Belaaouaj A, McCarthy R, Baumann M, et al. Mice lacking neutrophil elastase reveal impaired host defense against gram negative bacterial sepsis. Nat Med. 1998;4:615–8. 10.1038/nm0598-615. 9585238 10.1038/nm0598-615

[35] Chua F, Laurent GJ. Neutrophil elastase: mediator of extracellular matrix destruction and accumulation. Proc Am Thorac Soc. 2006;3:424–7. 10.1513/pats.200603-078AW. 16799086 10.1513/pats.200603-078AW

[36] Gao S, Zhu H, Zuo X, Luo H. Cathepsin G and its role in inflammation and autoimmune diseases. Arch Rheumatol. 2018;33:498–504. 10.5606/ArchRheumatol.2018.6595. 30874236 10.5606/ArchRheumatol.2018.6595 PMC6409175

[37] Aratani Y. Myeloperoxidase: its role for host defense, inflammation, and neutrophil function. Arch Biochem Biophys. 2018;640:47–52. 10.1016/j.abb.2018.01.004. 29336940 10.1016/j.abb.2018.01.004

[38] Klebanoff SJ. Myeloperoxidase: friend and foe. J Leukoc Biol. 2005;77:598–625. 10.1189/jlb.1204697. 15689384 10.1189/jlb.1204697

[39] Fuchs TA, Bhandari AA, Wagner DD. Histones induce rapid and profound thrombocytopenia in mice. Blood. 2011;118:3708–14. 10.1182/blood-2011-01-332676. 21700775 10.1182/blood-2011-01-332676 PMC3186342

[40] Papayannopoulos V. Neutrophil extracellular traps in immunity and disease. Nat Rev Immunol. 2018;18:134–47. 10.1038/nri.2017.105. 28990587 10.1038/nri.2017.105

[41] Islam M, Takeyama N. Role of neutrophil extracellular traps in health and disease pathophysiology: recent insights and advances. Int J Mol Sci. 2023;24:15805. 10.3390/ijms242115805. 37958788 10.3390/ijms242115805 PMC10649138

[42] Zhou Y, Xu Z, Liu Z. Impact of neutrophil extracellular traps on thrombosis formation: new findings and future perspective. Front Cell Infect Microbiol. 2022;12: 910908. 10.3389/fcimb.2022.910908. 35711663 10.3389/fcimb.2022.910908 PMC9195303

[43] Kawabata K, Hagio T, Matsumoto S, et al. Delayed neutrophil elastase inhibition prevents subsequent progression of acute lung injury induced by endotoxin inhalation in hamsters. Am J Respir Crit Care Med. 2000;161:2013–8. 10.1164/ajrccm.161.6.9904047. 10852782 10.1164/ajrccm.161.6.9904047

[44] Wang W, Su J, Kang W, et al. Neutrophil extracellular traps in autoimmune diseases: analysis of the knowledge map. Front Immunol. 2023;14:1095421. 10.3389/fimmu.2023.1095421. 36776836 10.3389/fimmu.2023.1095421 PMC9911519

[45] Wada J, Makino H. Innate immunity in diabetes and diabetic nephropathy. Nat Rev Nephrol. 2016;12:13–26. 10.1038/nrneph.2015.175. 26568190 10.1038/nrneph.2015.175

[46] Huang J, Hong W, Wan M, Zheng L. Molecular mechanisms and therapeutic target of NETosis in diseases. MedComm. 2022;3: e162. 10.1002/mco2.162. 36000086 10.1002/mco2.162 PMC9390875

[47] Wang L, Zhou W, Wang K, et al. Predictive value of circulating plasma mitochondrial DNA for sepsis in the emergency department: observational study based on the Sepsis-3 definition. BMC Emerg Med. 2020;20:25. 10.1186/s12873-020-00320-3. 32299369 10.1186/s12873-020-00320-3 PMC7164211

[48] Hotchkiss RS, Monneret G, Payen D. Sepsis-induced immunosuppression: from cellular dysfunctions to immunotherapy. Nat Rev Immunol. 2013;13:862–74. 10.1038/nri3552. 24232462 10.1038/nri3552 PMC4077177

[49] Galluzzi L, Vitale I, Aaronson SA, et al. Molecular mechanisms of cell death: recommendations of the Nomenclature Committee on Cell Death 2018. Cell Death Differ. 2018;25:486–541. 10.1038/s41418-017-0012-4. 29362479 10.1038/s41418-017-0012-4 PMC5864239

[50] Tian C, Wang K, Zhao M, et al. Extracellular vesicles participate in the pathogenesis of sepsis. Front Cell Infect Microbiol. 2022;12:1018692. 10.3389/fcimb.2022.1018692. 36579343 10.3389/fcimb.2022.1018692 PMC9791067

[51] Silk E, Zhao H, Weng H, Ma D. The role of extracellular histone in organ injury. Cell Death Dis. 2017;8: e2812. 10.1038/cddis.2017.52. 28542146 10.1038/cddis.2017.52 PMC5520745

[52] Iba T, Ogura H. Role of extracellular vesicles in the development of sepsis-induced coagulopathy. J Intensive Care. 2018;6:68. 10.1186/s40560-018-0340-6. 30377532 10.1186/s40560-018-0340-6 PMC6194680

[53] Chen R, Kang R, Fan X-G, Tang D. Release and activity of histone in diseases. Cell Death Dis. 2014;5: e1370. 10.1038/cddis.2014.337. 25118930 10.1038/cddis.2014.337 PMC4454312

[54] Huang H, Evankovich J, Yan W, et al. Endogenous histones function as alarmins in sterile inflammatory liver injury through Toll-like receptor 9 in mice. Hepatology. 2011;54:999–1008. 10.1002/hep.24501. 21721026 10.1002/hep.24501 PMC3213322

[55] Allam R, Kumar SVR, Darisipudi MN, Anders H-J. Extracellular histones in tissue injury and inflammation. J Mol Med. 2014;92:465–72. 10.1007/s00109-014-1148-z. 24706102 10.1007/s00109-014-1148-z

[56] Semeraro F, Ammollo CT, Morrissey JH, et al. Extracellular histones promote thrombin generation through platelet-dependent mechanisms: involvement of platelet TLR2 and TLR4. Blood. 2011;118:1952–61. 10.1182/blood-2011-03-343061. 21673343 10.1182/blood-2011-03-343061 PMC3158722

[57] Allam R, Darisipudi MN, Tschopp J, Anders H-J. Histones trigger sterile inflammation by activating the NLRP3 inflammasome. Eur J Immunol. 2013;43:3336–42. 10.1002/eji.201243224. 23964013 10.1002/eji.201243224

[58] Huang H, Chen H-W, Evankovich J, et al. Histones activate the NLRP3 inflammasome in Kupffer cells during sterile inflammatory liver injury. J Immunol. 2013;191:2665–79. 10.4049/jimmunol.1202733. 23904166 10.4049/jimmunol.1202733 PMC3777242

[59] Blevins HM, Xu Y, Biby S, Zhang S. The NLRP3 inflammasome pathway: a review of mechanisms and inhibitors for the treatment of inflammatory diseases. Front Aging Neurosci. 2022;14: 879021. 10.3389/fnagi.2022.879021. 35754962 10.3389/fnagi.2022.879021 PMC9226403

[60] Zhan X, Li Q, Xu G, et al. The mechanism of NLRP3 inflammasome activation and its pharmacological inhibitors. Front Immunol. 2022;13:1109938. 10.3389/fimmu.2022.1109938. 36741414 10.3389/fimmu.2022.1109938 PMC9889537

[61] Kelley N, Jeltema D, Duan Y, He Y. The NLRP3 inflammasome: an overview of mechanisms of activation and regulation. Int J Mol Sci. 2019;20:3328. 10.3390/ijms20133328. 31284572 10.3390/ijms20133328 PMC6651423

[62] Rathinam VAK, Fitzgerald KA. Inflammasome complexes: emerging mechanisms and effector functions. Cell. 2016;165:792–800. 10.1016/j.cell.2016.03.046. 27153493 10.1016/j.cell.2016.03.046 PMC5503689

[63] Marsman G, Zeerleder S, Luken BM. Extracellular histones, cell-free DNA, or nucleosomes: differences in immunostimulation. Cell Death Dis. 2016;7: e2518. 10.1038/cddis.2016.410. 27929534 10.1038/cddis.2016.410 PMC5261016

[64] Malamud M, Whitehead L, McIntosh A, et al. Recognition and control of neutrophil extracellular trap formation by MICL. Nature. 2024;633:442–50. 10.1038/s41586-024-07820-3. 39143217 10.1038/s41586-024-07820-3 PMC11390483

[65] Garcia-Romo GS, Caielli S, Vega B, et al. Netting neutrophils are major inducers of type I IFN production in pediatric systemic lupus erythematosus. Sci Transl Med. 2011;3:73ra20. 10.1126/scitranslmed.3001201. 21389264 10.1126/scitranslmed.3001201 PMC3143837

[66] McNab F, Mayer-Barber K, Sher A, et al. Type I interferons in infectious disease. Nat Rev Immunol. 2015;15:87–103. 10.1038/nri3787. 25614319 10.1038/nri3787 PMC7162685

[67] Tillack K, Breiden P, Martin R, Sospedra M. T lymphocyte priming by neutrophil extracellular traps links innate and adaptive immune responses. J Immunol. 2012;188:3150–9. 10.4049/jimmunol.1103414. 22351936 10.4049/jimmunol.1103414

[68] Shapouri-Moghaddam A, Mohammadian S, Vazini H, et al. Macrophage plasticity, polarization, and function in health and disease. J Cell Physiol. 2018;233:6425–40. 10.1002/jcp.26429. 29319160 10.1002/jcp.26429 PMC13482560

[69] Kimball AS, Obi AT, Diaz JA, Henke PK. The emerging role of NETs in venous thrombosis and immunothrombosis. Front Immunol. 2016;7:236. 10.3389/fimmu.2016.00236. 27446071 10.3389/fimmu.2016.00236 PMC4921471

[70] Kuang L, Wu Y, Shu J, et al. Pyroptotic macrophage-derived microvesicles accelerate formation of neutrophil extracellular traps via GSDMD-N-expressing mitochondrial transfer during sepsis. Int J Biol Sci. 2024;20:733–50. 10.7150/ijbs.87646. 38169726 10.7150/ijbs.87646 PMC10758106

[71] Gupta AK, Joshi MB, Philippova M, et al. Activated endothelial cells induce neutrophil extracellular traps and are susceptible to NETosis-mediated cell death. FEBS Lett. 2010;584:3193–7. 10.1016/j.febslet.2010.06.006. 20541553 10.1016/j.febslet.2010.06.006

[72] Iba T, Levy JH. Derangement of the endothelial glycocalyx in sepsis. J Thromb Haemost. 2019;17:283–94. 10.1111/jth.14371. 30582882 10.1111/jth.14371

[73] Wang H, Wang C, Zhao M-H, Chen M. Neutrophil extracellular traps can activate alternative complement pathways. Clin Exp Immunol. 2015;181:518–27. 10.1111/cei.12654. 25963026 10.1111/cei.12654 PMC4557387

[74] De Bont CM, Boelens WC, Pruijn GJM. NETosis, complement, and coagulation: a triangular relationship. Cell Mol Immunol. 2019;16:19–27. 10.1038/s41423-018-0024-0. 29572545 10.1038/s41423-018-0024-0 PMC6318284

[75] Fattahi F, Grailer JJ, Jajou L, et al. Organ distribution of histones after intravenous infusion of FITC histones or after sepsis. Immunol Res. 2015;61:177–86. 10.1007/s12026-015-8628-2. 25680340 10.1007/s12026-015-8628-2 PMC4339508

[76] Farrera C, Fadeel B. Macrophage clearance of neutrophil extracellular traps is a silent process. J Immunol. 2013;191:2647–56. 10.4049/jimmunol.1300436. 23904163 10.4049/jimmunol.1300436

[77] Kapoor S, Opneja A, Nayak L. The role of neutrophils in thrombosis. Thromb Res. 2018;170:87–96. 10.1016/j.thromres.2018.08.005. 30138777 10.1016/j.thromres.2018.08.005 PMC6174090

[78] Carestia A, Rivadeneyra L, Romaniuk MA, et al. Functional responses and molecular mechanisms involved in histone-mediated platelet activation. Thromb Haemost. 2013;110:1035–45. 10.1160/TH13-02-0174. 23965842 10.1160/TH13-02-0174

[79] Semeraro F, Ammollo CT, Esmon NL, Esmon CT. Histones induce phosphatidylserine exposure and a procoagulant phenotype in human red blood cells. J Thromb Haemost. 2014;12:1697–702. 10.1111/jth.12677. 25069624 10.1111/jth.12677 PMC4194154

[80] Ammollo CT, Semeraro F, Xu J, et al. Extracellular histones increase plasma thrombin generation by impairing thrombomodulin-dependent protein C activation. J Thromb Haemost. 2011;9:1795–803. 10.1111/j.1538-7836.2011.04422.x. 21711444 10.1111/j.1538-7836.2011.04422.x

[81] Behzadifard M, Soleimani M. NETosis and SARS-COV-2 infection related thrombosis: a narrative review. Thromb J. 2022;20:13. 10.1186/s12959-022-00375-1. 35354492 10.1186/s12959-022-00375-1 PMC8965217

[82] Gould TJ, Lysov Z, Liaw PC. Extracellular DNA and histones: double-edged swords in immunothrombosis. J Thromb Haemost. 2015;13:S82–91. 10.1111/jth.12977. 26149054 10.1111/jth.12977

[83] Maas C, Renné T. Coagulation factor XII in thrombosis and inflammation. Blood. 2018;131:1903–9. 10.1182/blood-2017-04-569111. 29483100 10.1182/blood-2017-04-569111

[84] Yong J, Abrams ST, Wang G, Toh C-H. Cell-free histones and the cell-based model of coagulation. J Thromb Haemost. 2023;21:1724–36. 10.1016/j.jtha.2023.04.018. 37116754 10.1016/j.jtha.2023.04.018

[85] Hoffman M, Monroe DM. A cell-based model of hemostasis. Thromb Haemost. 2001;85:958–65. 11434702

[86] Fuchs TA, Brill A, Duerschmied D, et al. Extracellular DNA traps promote thrombosis. Proc Natl Acad Sci. 2010;107:15880–5. 10.1073/pnas.1005743107. 20798043 10.1073/pnas.1005743107 PMC2936604

[87] Martinod K, Wagner DD. Thrombosis: tangled up in NETs. Blood. 2014;123:2768–76. 10.1182/blood-2013-10-463646. 24366358 10.1182/blood-2013-10-463646 PMC4007606

[88] Saffarzadeh M, Juenemann C, Queisser MA, et al. Neutrophil extracellular traps directly induce epithelial and endothelial cell death: a predominant role of histones. PLoS ONE. 2012;7: e32366. 10.1371/journal.pone.0032366. 22389696 10.1371/journal.pone.0032366 PMC3289648

[89] Yamamichi S, Fujiwara Y, Kikuchi T, et al. Extracellular histone induces plasma hyaluronan-binding protein (factor VII activating protease) activation in vivo. Biochem Biophys Res Commun. 2011;409:483–8. 10.1016/j.bbrc.2011.05.030. 21600885 10.1016/j.bbrc.2011.05.030

[90] Yang X, Li L, Liu J, et al. Extracellular histones induce tissue factor expression in vascular endothelial cells via TLR and activation of NF-κB and AP-1. Thromb Res. 2016;137:211–8. 10.1016/j.thromres.2015.10.012. 26476743 10.1016/j.thromres.2015.10.012

[91] Michels A, Albánez S, Mewburn J, et al. Histones link inflammation and thrombosis through the induction of Weibel-Palade body exocytosis. J Thromb Haemost. 2016;14:2274–86. 10.1111/jth.13493. 27589692 10.1111/jth.13493

[92] Fousert E, Toes R, Desai J. Neutrophil extracellular traps (NETs) take the central stage in driving autoimmune responses. Cells. 2020;9:915. 10.3390/cells9040915. 32276504 10.3390/cells9040915 PMC7226846

[93] Iba T, Levy JH, Connors JM, et al. The unique characteristics of COVID-19 coagulopathy. Crit Care. 2020;24:360. 10.1186/s13054-020-03077-0. 32552865 10.1186/s13054-020-03077-0 PMC7301352

[94] Cheng Z, Abrams ST, Alhamdi Y, et al. Circulating histones are major mediators of multiple organ dysfunction syndrome in acute critical illnesses. Crit Care Med. 2019;47:e677–84. 10.1097/CCM.0000000000003839. 31162199 10.1097/CCM.0000000000003839

[95] McDonald B, Davis RP, Kim S-J, et al. Platelets and neutrophil extracellular traps collaborate to promote intravascular coagulation during sepsis in mice. Blood. 2017;129:1357–67. 10.1182/blood-2016-09-741298. 28073784 10.1182/blood-2016-09-741298 PMC5345735

[96] Middleton EA, He X-Y, Denorme F, et al. Neutrophil extracellular traps contribute to immunothrombosis in COVID-19 acute respiratory distress syndrome. Blood. 2020;136:1169–79. 10.1182/blood.2020007008. 32597954 10.1182/blood.2020007008 PMC7472714

[97] Knight JS, Meng H, Coit P, et al. Activated signature of antiphospholipid syndrome neutrophils reveals potential therapeutic target. JCI Insight. 2017;2: e93897. 10.1172/jci.insight.93897. 28931754 10.1172/jci.insight.93897 PMC5621930

[98] Abrams ST, Morton B, Alhamdi Y, et al. A novel assay for neutrophil extracellular trap formation independently predicts disseminated intravascular coagulation and mortality in critically ill patients. Am J Respir Crit Care Med. 2019;200:869–80. 10.1164/rccm.201811-2111OC. 31162936 10.1164/rccm.201811-2111OC PMC6812439

[99] Costello RA, Leslie SW, Nehring SM (2024) Disseminated intravascular coagulation. In: StatPearls. StatPearls Publishing, Treasure Island (FL) 28722864

[100] Barnes BJ, Adrover JM, Baxter-Stoltzfus A, et al. Targeting potential drivers of COVID-19: neutrophil extracellular traps. J Exp Med. 2020;217: e20200652. 10.1084/jem.20200652. 32302401 10.1084/jem.20200652 PMC7161085

[101] Carvelli J, Demaria O, Vély F, et al. Association of COVID-19 inflammation with activation of the C5a–C5aR1 axis. Nature. 2020;588:146–50. 10.1038/s41586-020-2600-6. 32726800 10.1038/s41586-020-2600-6 PMC7116884

[102] Loh JT, Zhang B, Teo JKH, et al. Mechanism for the attenuation of neutrophil and complement hyperactivity by MSC exosomes. Cytotherapy. 2022;24:711–9. 10.1016/j.jcyt.2021.12.003. 35177337 10.1016/j.jcyt.2021.12.003 PMC8843421

[103] Brouwer S, Rivera-Hernandez T, Curren BF, et al. Pathogenesis, epidemiology and control of group A *Streptococcus* infection. Nat Rev Microbiol. 2023;21:431–47. 10.1038/s41579-023-00865-7. 36894668 10.1038/s41579-023-00865-7 PMC9998027

[104] Berends ETM, Horswill AR, Haste NM, et al. Nuclease expression by *Staphylococcus aureus* facilitates escape from neutrophil extracellular traps. J Innate Immun. 2010;2:576–86. 10.1159/000319909. 20829609 10.1159/000319909 PMC2982853

[105] Derré-Bobillot A, Cortes-Perez NG, Yamamoto Y, et al. Nuclease A (Gbs0661), an extracellular nuclease of *Streptococcus agalactiae*, attacks the neutrophil extracellular traps and is needed for full virulence. Mol Microbiol. 2013;89:518–31. 10.1111/mmi.12295. 23772975 10.1111/mmi.12295

[106] Buchanan JT, Simpson AJ, Aziz RK, et al. DNase expression allows the pathogen group A *Streptococcus* to escape killing in neutrophil extracellular traps. Curr Biol. 2006;16:396–400. 10.1016/j.cub.2005.12.039. 16488874 10.1016/j.cub.2005.12.039

[107] Thammavongsa V, Missiakas DM, Schneewind O. *Staphylococcus aureus* degrades neutrophil extracellular traps to promote immune cell death. Science. 2013;342:863–6. 10.1126/science.1242255. 24233725 10.1126/science.1242255 PMC4026193

[108] Howden BP, Giulieri SG, Wong Fok Lung T, et al. *Staphylococcus aureus* host interactions and adaptation. Nat Rev Microbiol. 2023;21:380–95. 10.1038/s41579-023-00852-y. 36707725 10.1038/s41579-023-00852-y PMC9882747

[109] Walker MJ, Hollands A, Sanderson-Smith ML, et al. DNase Sda1 provides selection pressure for a switch to invasive group A streptococcal infection. Nat Med. 2007;13:981–5. 10.1038/nm1612. 17632528 10.1038/nm1612

[110] Lappann M, Danhof S, Guenther F, et al. In vitro resistance mechanisms of *Neisseria meningitidis* against neutrophil extracellular traps. Mol Microbiol. 2013;89:433–49. 10.1111/mmi.12288. 23750848 10.1111/mmi.12288

[111] Wartha F, Beiter K, Albiger B, et al. Capsule and d-alanylated lipoteichoic acids protect *Streptococcus pneumoniae* against neutrophil extracellular traps. Cell Microbiol. 2007;9:1162–71. 10.1111/j.1462-5822.2006.00857.x. 17217430 10.1111/j.1462-5822.2006.00857.x

[112] Beiter K, Wartha F, Albiger B, et al. An endonuclease allows *Streptococcus pneumoniae* to escape from neutrophil extracellular traps. Curr Biol. 2006;16:401–7. 10.1016/j.cub.2006.01.056. 16488875 10.1016/j.cub.2006.01.056

[113] Jhelum H, Sori H, Sehgal D. A novel extracellular vesicle-associated endodeoxyribonuclease helps *Streptococcus pneumoniae* evade neutrophil extracellular traps and is required for full virulence. Sci Rep. 2018;8:7985. 10.1038/s41598-018-25865-z. 29789571 10.1038/s41598-018-25865-z PMC5964101

[114] Nel JG, Theron AJ, Durandt C, et al. Pneumolysin activates neutrophil extracellular trap formation. Clin Exp Immunol. 2016;184:358–67. 10.1111/cei.12766. 26749379 10.1111/cei.12766 PMC4872380

[115] Spinner JL, Winfree S, Starr T, et al. *Yersinia pestis* survival and replication within human neutrophil phagosomes and uptake of infected neutrophils by macrophages. J Leukoc Biol. 2013;95:389–98. 10.1189/jlb.1112551. 24227798 10.1189/jlb.1112551 PMC3923079

[116] Palace SG, Vitseva O, Proulx MK, et al. *Yersinia pestis* escapes entrapment in thrombi by targeting platelet function. J Thromb Haemost. 2020;18:3236–48. 10.1111/jth.15065. 33470041 10.1111/jth.15065 PMC8040536

[117] Eby JC, Gray MC, Hewlett EL. Cyclic AMP-mediated suppression of neutrophil extracellular trap formation and apoptosis by the *Bordetella pertussis* adenylate cyclase toxin. Infect Immun. 2014;82:5256–69. 10.1128/IAI.02487-14. 25287922 10.1128/IAI.02487-14 PMC4249293

[118] Jesaitis AJ, Franklin MJ, Berglund D, et al. Compromised host defense on *Pseudomonas aeruginosa* biofilms: characterization of neutrophil and biofilm interactions. J Immunol. 2003;171:4329–39. 10.4049/jimmunol.171.8.4329. 14530358 10.4049/jimmunol.171.8.4329

[119] Thammavongsa V, Kim HK, Missiakas D, Schneewind O. Staphylococcal manipulation of host immune responses. Nat Rev Microbiol. 2015;13:529–43. 10.1038/nrmicro3521. 26272408 10.1038/nrmicro3521 PMC4625792

[120] Speziale P, Pietrocola G. *Staphylococcus aureus* induces neutrophil extracellular traps (NETs) and neutralizes their bactericidal potential. Comput Struct Biotechnol J. 2021;19:3451–7. 10.1016/j.csbj.2021.06.012. 34194670 10.1016/j.csbj.2021.06.012 PMC8220102

[121] Moscoso M, García E, López R. Biofilm formation by *Streptococcus pneumoniae* : role of choline, extracellular DNA, and capsular polysaccharide in microbial accretion. J Bacteriol. 2006;188:7785–95. 10.1128/JB.00673-06. 16936041 10.1128/JB.00673-06 PMC1636320

[122] Birnberg-Weiss F, Castillo LA, Pittaluga JR, et al. Modulation of neutrophil extracellular traps release by *Klebsiella pneumoniae*. J Leukoc Biol. 2021;109:245–56. 10.1002/JLB.4MA0620-099R. 32640486 10.1002/JLB.4MA0620-099R

[123] Flemming H-C, Wingender J. The biofilm matrix. Nat Rev Microbiol. 2010;8:623–33. 10.1038/nrmicro2415. 20676145 10.1038/nrmicro2415

[124] Nauseef WM. How human neutrophils kill and degrade microbes: an integrated view. Immunol Rev. 2007;219:88–102. 10.1111/j.1600-065X.2007.00550.x. 17850484 10.1111/j.1600-065X.2007.00550.x

[125] Happonen L, Collin M. Immunomodulating enzymes from *Streptococcus pyogenes*—in pathogenesis, as biotechnological tools, and as biological drugs. Microorganisms. 2024;12:200. 10.3390/microorganisms12010200. 38258026 10.3390/microorganisms12010200 PMC10818452

[126] Skopelja-Gardner S, Theprungsirikul J, Lewis KA, et al. Regulation of *Pseudomonas aeruginosa*-mediated neutrophil extracellular traps. Front Immunol. 2019;10:1670. 10.3389/fimmu.2019.01670. 31379861 10.3389/fimmu.2019.01670 PMC6657737

[127] Borkute RR, Woelke S, Pei G, Dorhoi A. Neutrophils in tuberculosis: cell biology, cellular networking and multitasking in host defense. Int J Mol Sci. 2021;22:4801. 10.3390/ijms22094801. 33946542 10.3390/ijms22094801 PMC8125784

[128] Li P, Li M, Lindberg MR, et al. PAD4 is essential for antibacterial innate immunity mediated by neutrophil extracellular traps. J Exp Med. 2010;207:1853–62. 10.1084/jem.20100239. 20733033 10.1084/jem.20100239 PMC2931169

[129] Knight JS, Zhao W, Luo W, et al. Peptidylarginine deiminase inhibition is immunomodulatory and vasculoprotective in murine lupus. J Clin Invest. 2013;123:2981–93. 10.1172/JCI67390. 23722903 10.1172/JCI67390 PMC3696545

[130] Knight JS, Luo W, O’Dell AA, et al. Peptidylarginine deiminase inhibition reduces vascular damage and modulates innate immune responses in murine models of atherosclerosis. Circ Res. 2014;114:947–56. 10.1161/CIRCRESAHA.114.303312. 24425713 10.1161/CIRCRESAHA.114.303312 PMC4185401

[131] Biron BM, Chung C-S, O’Brien XM, et al. Cl-amidine prevents histone 3 citrullination and neutrophil extracellular trap formation, and improves survival in a murine sepsis model. J Innate Immun. 2017;9:22–32. 10.1159/000448808. 27622642 10.1159/000448808 PMC5219946

[132] Lewis HD, Liddle J, Coote JE, et al. Inhibition of PAD4 activity is sufficient to disrupt mouse and human NET formation. Nat Chem Biol. 2015;11:189–91. 10.1038/nchembio.1735. 25622091 10.1038/nchembio.1735 PMC4397581

[133] Altenhöfer S, Radermacher KA, Kleikers PWM, et al. Evolution of NADPH oxidase inhibitors: selectivity and mechanisms for target engagement. Antioxid Redox Signal. 2015;23:406–27. 10.1089/ars.2013.5814. 24383718 10.1089/ars.2013.5814 PMC4543484

[134] Hirano K, Chen WS, Chueng ALW, et al. Discovery of GSK2795039, a novel small molecule NADPH oxidase 2 inhibitor. Antioxid Redox Signal. 2015;23:358–74. 10.1089/ars.2014.6202. 26135714 10.1089/ars.2014.6202 PMC4545375

[135] Cha JJ, Min HS, Kim KT, et al. APX-115, a first-in-class pan-NADPH oxidase (Nox) inhibitor, protects db/db mice from renal injury. Lab Investig J Tech Methods Pathol. 2017;97:419–31. 10.1038/labinvest.2017.2. 10.1038/labinvest.2017.2 28165467

[136] Dorotea D, Kwon G, Lee JH, et al. A pan-NADPH oxidase inhibitor ameliorates kidney injury in type 1 diabetic rats. Pharmacology. 2018;102:180–9. 10.1159/000491398. 30099457 10.1159/000491398

[137] McCoy K, Hamilton S, Johnson C. Effects of 12-week administration of dornase alfa in patients with advanced cystic fibrosis lung disease. Pulmozyme Study Group Chest. 1996;110:889–95. 10.1378/chest.110.4.889. 10.1378/chest.110.4.889 8874241

[138] Biermann MH, Veissi S, Maueröder C, et al. The role of dead cell clearance in the etiology and pathogenesis of systemic lupus erythematosus: dendritic cells as potential targets. Expert Rev Clin Immunol. 2014;10:1151–64. 10.1586/1744666X.2014.944162. 25081199 10.1586/1744666X.2014.944162

[139] Reutelingsperger CPM, Gijbels MJ, Spronk H, et al. M6229 protects against extracellular-histone-induced liver injury, kidney dysfunction, and mortality in a rat model of acute hyperinflammation. Int J Mol Sci. 2024;25:1376. 10.3390/ijms25031376. 38338654 10.3390/ijms25031376 PMC10855969

[140] Jucaite A, Svenningsson P, Rinne JO, et al. Effect of the myeloperoxidase inhibitor AZD3241 on microglia: a PET study in Parkinson’s disease. Brain. 2015;138:2687–700. 10.1093/brain/awv184. 26137956 10.1093/brain/awv184

[141] Zeiher BG, Matsuoka S, Kawabata K, Repine JE. Neutrophil elastase and acute lung injury: prospects for sivelestat and other neutrophil elastase inhibitors as therapeutics. Crit Care Med. 2002;30:S281–7. 10.1097/00003246-200205001-00018. 12004249 10.1097/00003246-200205001-00018

[142] Kawasaki Y, Aikawa N. Clinical utility of the neutrophil elastase inhibitor sivelestat for the treatment of acute respiratory distress syndrome. Ther Clin Risk Manag. 2014;10:621–9. 10.2147/TCRM.S65066. 25120368 10.2147/TCRM.S65066 PMC4130327

[143] Tagami T, Tosa R, Omura M, et al. Effect of a selective neutrophil elastase inhibitor on mortality and ventilator-free days in patients with increased extravascular lung water: a post hoc analysis of the PiCCO Pulmonary Edema Study. J Intensive Care. 2014;2:67. 10.1186/s40560-014-0067-y. 25705423 10.1186/s40560-014-0067-y PMC4336272

[144] Aswani A, Genkin D, Surkov K, et al. Selective cfDNA/NETs apheresis with NucleoCapture™ in a prolonged clinically relevant porcine intensive care sepsis model. Intensive Care Med Exp. 2023;11: 001002.

[145] Aswani A, Genkin D, Surkov K, et al. Therapeutic removal of cfDNA/NETs using NucleoCapture™ apheresis in a porcine intensive care sepsis model: a blinded randomised controlled trial. Intensive Care Med Exp. 2023;11: 000806. 10.1186/s40635-023-00546-y.

[146] Aswani A, Abramovsky S, Afanasieva MI, et al. Safety and performance of the NucleoCapture™ Column for selective therapeutic removal of cfDNA/NETs in patients with sepsis. Intensive Care Med Exp. 2023;11: 000679. 10.1186/s40635-023-00546-y.

[147] Rohrbach AS, Slade DJ, Thompson PR, Mowen KA. Activation of PAD4 in NET formation. Front Immunol. 2012;3:360. 10.3389/fimmu.2012.00360. 23264775 10.3389/fimmu.2012.00360 PMC3525017

[148] Yuzhalin AE. Citrullination in cancer. Cancer Res. 2019;79:1274–84. 10.1158/0008-5472.CAN-18-2797. 30894374 10.1158/0008-5472.CAN-18-2797

[149] Björnsdottir H, Welin A, Michaëlsson E, et al. Neutrophil NET formation is regulated from the inside by myeloperoxidase-processed reactive oxygen species. Free Radic Biol Med. 2015;89:1024–35. 10.1016/j.freeradbiomed.2015.10.398. 26459032 10.1016/j.freeradbiomed.2015.10.398

[150] Singel KL, Segal BH. NOX2-dependent regulation of inflammation. Clin Sci. 2016;130:479–90. 10.1042/CS20150660. 10.1042/CS20150660 PMC5513728 26888560

[151] Okur HK, Yalcin K, Tastan C, et al. Preliminary report of in vitro and in vivo effectiveness of dornase alfa on SARS-CoV-2 infection. New Microbes New Infect. 2020;37: 100756. 10.1016/j.nmni.2020.100756. 32922804 10.1016/j.nmni.2020.100756 PMC7476504

[152] Mai SHC, Khan M, Dwivedi DJ, et al. Delayed but not early treatment with DNase reduces organ damage and improves outcome in a murine model of sepsis. Shock. 2015;44:166–72. 10.1097/SHK.0000000000000396. 26009820 10.1097/SHK.0000000000000396

[153] Lefrançais E, Mallavia B, Zhuo H, et al. Maladaptive role of neutrophil extracellular traps in pathogen-induced lung injury. JCI Insight. 2018;3: e98178. 10.1172/jci.insight.98178. 29415887 10.1172/jci.insight.98178 PMC5821185

[154] Shak S, Capon DJ, Hellmiss R, et al. Recombinant human DNase I reduces the viscosity of cystic fibrosis sputum. Proc Natl Acad Sci U S A. 1990;87:9188–92. 10.1073/pnas.87.23.9188. 2251263 10.1073/pnas.87.23.9188 PMC55129

[155] Mogayzel PJ, Naureckas ET, Robinson KA, et al. Cystic fibrosis pulmonary guidelines: chronic medications for maintenance of lung health. Am J Respir Crit Care Med. 2013;187:680–9. 10.1164/rccm.201207-1160OE. 23540878 10.1164/rccm.201207-1160oe

[156] Várady CBS, Oliveira AC, Monteiro RQ, Gomes T. Recombinant human DNase I for the treatment of cancer-associated thrombosis: a pre-clinical study. Thromb Res. 2021;203:131–7. 10.1016/j.thromres.2021.04.028. 34015562 10.1016/j.thromres.2021.04.028

[157] Sharma S, Hofbauer TM, Ondracek AS, et al. Neutrophil extracellular traps promote fibrous vascular occlusions in chronic thrombosis. Blood. 2021;137:1104–16. 10.1182/blood.2020005861. 33512471 10.1182/blood.2020005861

[158] Brill A, Fuchs TA, Savchenko AS, et al. Neutrophil extracellular traps promote deep vein thrombosis in mice. J Thromb Haemost. 2012;10:136–44. 10.1111/j.1538-7836.2011.04544.x. 22044575 10.1111/j.1538-7836.2011.04544.x PMC3319651

[159] Ngo ATP, Gollomp K. Building a better NET: Neutrophil extracellular trap targeted therapeutics in the treatment of infectious and inflammatory disorders. Res Pract Thromb Haemost. 2022;6: e12808. 10.1002/rth2.12808.

[160] Wang H, Kim SJ, Lei Y, et al. Neutrophil extracellular traps in homeostasis and disease. Signal Transduct Target Ther. 2024;9:235. 10.1038/s41392-024-01933-x. 39300084 10.1038/s41392-024-01933-x PMC11415080

[161] Jiménez-Alcázar M, Rangaswamy C, Panda R, et al. Host DNases prevent vascular occlusion by neutrophil extracellular traps. Science. 2017;358:1202–6. 10.1126/science.aam8897. 29191910 10.1126/science.aam8897

[162] Leffler J, Martin M, Gullstrand B, et al. Neutrophil extracellular traps that are not degraded in systemic lupus erythematosus activate complement exacerbating the disease. J Immunol. 2012;188:3522–31. 10.4049/jimmunol.1102404. 22345666 10.4049/jimmunol.1102404

[163] Bossi F, Tripodo C, Rizzi L, et al. C1q as a unique player in angiogenesis with therapeutic implication in wound healing. Proc Natl Acad Sci U S A. 2014;111:4209–14. 10.1073/pnas.1311968111. 24591625 10.1073/pnas.1311968111 PMC3964125

[164] Wagener JS, Kupfer O. Dornase alfa (Pulmozyme): Curr Opin Pulm Med. 2012;18:609–14. 10.1097/MCP.0b013e328358d51f. 22990660 10.1097/MCP.0b013e328358d51f

[165] Yang C, Montgomery M. Dornase alfa for cystic fibrosis. Cochrane Database Syst Rev. 2018. 10.1002/14651858.CD001127.pub4. 30187450 10.1002/14651858.CD001127.pub4 PMC6513278

[166] Papayannopoulos V, Metzler KD, Hakkim A, Zychlinsky A. Neutrophil elastase and myeloperoxidase regulate the formation of neutrophil extracellular traps. J Cell Biol. 2010;191:677–91. 10.1083/jcb.201006052. 20974816 10.1083/jcb.201006052 PMC3003309

[167] Abrams ST, Zhang N, Manson J, et al. Circulating histones are mediators of trauma-associated lung injury. Am J Respir Crit Care Med. 2013;187:160–9. 10.1164/rccm.201206-1037OC. 23220920 10.1164/rccm.201206-1037OC PMC3570656

[168] ClinicalTrials.gov Intravenously administered M6229 in critically ill sepsis patients. https://clinicaltrials.gov/study/NCT05208112?a=4. Accessed 6 Aug 2024

[169] Morad H, Luqman S, Tan C-H, et al. TRPM2 ion channels steer neutrophils towards a source of hydrogen peroxide. Sci Rep. 2021;11:9339. 10.1038/s41598-021-88224-5. 33927223 10.1038/s41598-021-88224-5 PMC8085234

[170] Metzler KD, Fuchs TA, Nauseef WM, et al. Myeloperoxidase is required for neutrophil extracellular trap formation: implications for innate immunity. Blood. 2011;117:953–9. 10.1182/blood-2010-06-290171. 20974672 10.1182/blood-2010-06-290171 PMC3035083

[171] Teng N, Maghzal GJ, Talib J, et al. The roles of myeloperoxidase in coronary artery disease and its potential implication in plaque rupture. Redox Rep. 2017;22:51–73. 10.1080/13510002.2016.1256119. 27884085 10.1080/13510002.2016.1256119 PMC6837458

[172] Healy LD, Puy C, Fernández JA, et al. Activated protein C inhibits neutrophil extracellular trap formation in vitro and activation in vivo. J Biol Chem. 2017;292:8616–29. 10.1074/jbc.M116.768309. 28408624 10.1074/jbc.M116.768309 PMC5448091

[173] Yang T, Peng J, Zhang Z, et al. Emerging therapeutic strategies targeting extracellular histones for critical and inflammatory diseases: an updated narrative review. Front Immunol. 2024;15:1438984. 10.3389/fimmu.2024.1438984. 39206200 10.3389/fimmu.2024.1438984 PMC11349558

[174] Huckriede JB, Beurskens DMH, Wildhagen KCCA, et al. Design and characterization of novel activated protein C variants for the proteolysis of cytotoxic extracellular histone H3. J Thromb Haemost. 2023;21:3557–67. 10.1016/j.jtha.2023.08.023. 37657561 10.1016/j.jtha.2023.08.023

[175] Polverino E, Rosales-Mayor E, Dale GE, et al. The role of neutrophil elastase inhibitors in lung diseases. Chest. 2017;152:249–62. 10.1016/j.chest.2017.03.056. 28442313 10.1016/j.chest.2017.03.056

[176] Mitsios A, Arampatzioglou A, Arelaki S, et al. NETopathies? Unraveling the dark side of old diseases through neutrophils. Front Immunol. 2017. 10.3389/fimmu.2016.00678. 28123386 10.3389/fimmu.2016.00678 PMC5225098

[177] Stoimenou M, Tzoros G, Skendros P, Chrysanthopoulou A. Methods for the assessment of NET formation: from neutrophil biology to translational research. Int J Mol Sci. 2022;23:15823. 10.3390/ijms232415823. 36555464 10.3390/ijms232415823 PMC9781911

[178] Su F, Moreau A, Savi M, et al. Circulating nucleosomes as a novel biomarker for sepsis: a scoping review. Biomedicines. 2024;12:1385. 10.3390/biomedicines12071385. 39061959 10.3390/biomedicines12071385 PMC11273886

[179] Kasprzycka W, Homa-Mlak I, Mlak R, Małecka-Massalska T. Direct and indirect methods of evaluating the NETosis process. J Pre-Clin Clin Res. 2019;13:50–6. 10.26444/jpccr/105563.

[180] Xu X, Wu Y, Xu S, et al. Clinical significance of neutrophil extracellular traps biomarkers in thrombosis. Thromb J. 2022;20:63. 10.1186/s12959-022-00421-y. 36224604 10.1186/s12959-022-00421-y PMC9555260

[181] Donkel SJ, Wolters FJ, Ikram MA, de Maat MPM. Circulating myeloperoxidase (MPO)-DNA complexes as marker for neutrophil extracellular traps (NETs) levels and the association with cardiovascular risk factors in the general population. PLoS ONE. 2021;16: e0253698. 10.1371/journal.pone.0253698. 34379628 10.1371/journal.pone.0253698 PMC8357174

[182] Thålin C, Aguilera K, Hall NW, et al. Quantification of citrullinated histones: development of an improved assay to reliably quantify nucleosomal H3Cit in human plasma. J Thromb Haemost. 2020;18:2732–43. 10.1111/jth.15003. 32654410 10.1111/jth.15003 PMC8722705

[183] Maruchi Y, Tsuda M, Mori H, et al. Plasma myeloperoxidase-conjugated DNA level predicts outcomes and organ dysfunction in patients with septic shock. Crit Care. 2018;22:176. 10.1186/s13054-018-2109-7. 30005596 10.1186/s13054-018-2109-7 PMC6045839

[184] Bonaventura A, Carbone F, Vecchié A, et al. The role of resistin and myeloperoxidase in severe sepsis and septic shock: results from the ALBIOS trial. Eur J Clin Invest. 2020;50: e13333. 10.1111/eci.13333. 32585739 10.1111/eci.13333

[185] Laridan E, Martinod K, De Meyer SF. Neutrophil extracellular traps in arterial and venous thrombosis. Semin Thromb Hemost. 2019;45:86–93. 10.1055/s-0038-1677040. 30634198 10.1055/s-0038-1677040

[186] Fuchs TA, Brill A, Wagner DD. Neutrophil extracellular trap (NET) impact on deep vein thrombosis. Arterioscler Thromb Vasc Biol. 2012;32:1777–83. 10.1161/ATVBAHA.111.242859. 22652600 10.1161/ATVBAHA.111.242859 PMC3495595

[187] Yipp BG, Kubes P. NETosis: how vital is it? Blood. 2013;122:2784–94. 10.1182/blood-2013-04-457671. 24009232 10.1182/blood-2013-04-457671

[188] Clark SR, Ma AC, Tavener SA, et al. Platelet TLR4 activates neutrophil extracellular traps to ensnare bacteria in septic blood. Nat Med. 2007;13:463–9. 10.1038/nm1565. 17384648 10.1038/nm1565

[189] Yousefi S, Mihalache C, Kozlowski E, et al. Viable neutrophils release mitochondrial DNA to form neutrophil extracellular traps. Cell Death Differ. 2009;16:1438–44. 10.1038/cdd.2009.96. 19609275 10.1038/cdd.2009.96

